# A new scenario of pathogen-microbiota interactions involving the oomycete *Plasmopara viticola*

**DOI:** 10.1093/femsec/fiaf111

**Published:** 2025-11-06

**Authors:** Paola Fournier, Lucile Pellan, Julie Aubert, Patrice This, Corinne Vacher

**Affiliations:** INRAE, Bordeaux Sciences Agro, ISVV, SAVE, 33882Villenave-d’Ornon, France; Ecophysiologie et Génomique Fonctionnelle de la Vigne (EGFV), Université de Bordeaux, Bordeaux Sciences Agro, INRAE, ISVV, 33882 Villenave d’Ornon, France; Université Paris-Saclay, AgroParisTech, INRAE, UMR MIA-Paris-Saclay, 91120 Palaiseau, France; UMR AGAP Institut, Univ Montpellier, CIRAD, INRAE, Institut Agro, 34398 Montpellier, France; INRAE, Bordeaux Sciences Agro, ISVV, SAVE, 33882Villenave-d’Ornon, France

**Keywords:** biocontrol, community ecology, Grapevine downy mildew, host–microbiota–pathogen interactions, microbial community assembly

## Abstract

A key question in microbial ecology is how the microbiota regulates host invasion by pathogens. Several ecological theories link the diversity, abundance and assembly processes of the microbiota with its resistance to invasion, but the specific properties of microbial communities that confer protection to the host are poorly understood.

We addressed this question for the oomycete *Plasmopara viticola*, the causal agent of grapevine downy mildew. Using state-of-the-art microbial ecology methods, we compared microbial communities associated with asymptomatic and symptomatic leaf tissues to elucidate pathogen-microbiota interactions.

Despite visible symptoms, *P. viticola* infection induced only subtle changes in microbial community composition. Symptomatic tissues showed enrichment in basidiomycete yeasts and *Bacillus* species, both known for their biocontrol activity, and exhibited a higher degree of determinism in community assembly processes. Asymptomatic tissues hosted more diverse microbiota, but lacked consistent associations with known biocontrol agents. Instead, they were often associated with other airborne grapevine pathogens.

These findings suggest a novel interaction scenario: upon infection, *P. viticola* reshapes locally the leaf microbiota, excluding other pathogens and selecting for beneficial microbes. Although further studies are needed to uncover the underlying mechanisms, these findings underscore the relevance of targeting disease lesions in the search for protective microbial consortia.

## Teaser

The development of downy mildew in grapevine leaf tissues tends to exclude other pathogens and promotes the local recruitment of specific microbial taxa with biocontrol activity, such as basidiomycetous yeasts.

## Introduction

The role of the microbiota in the emergence and spread of diseases has long been overlooked. However, the intricate interactions between hosts, pathogens and the microbiota are now central to disease research, as host resistance to diseases is, at least in part, driven by the composition of the microbiota and the occupation of the microbial niche (Vannier et al. 2019, Liu et al. 2020, Ping et al. 2024). This growing awareness has spurred the development of new concepts, such as disease ecology and the pathobiome (Vayssier-Taussat et al. 2014, Bass et al. 2019), and more recently, the concept of a protective microbiota (Goossens et al. 2023). Central to this field are questions regarding how the microbiota regulates invasion by pathogens (Teixeira et al. 2019, Li et al. 2021) and how the host maintains microbiota homeostasis (Hacquard et al. 2017, Karasov et al. 2020, Paasch and He 2021). Addressing these questions is key to fighting diseases by harnessing the microbiota, whether through preventive or curative inoculation of microorganisms; the use of metabolites that steer microbial ecosystem functioning; or the modulation of host physiology, defense mechanisms, and environmental conditions (Busby et al. 2017, Compant et al. 2025).

Over the past decade, numerous studies have compared the microbial communities of visually healthy tissues to those of tissues infected by various plant pathogens, aiming to identify the properties of the microbiota that promote or inhibit disease (Jakuschkin et al. 2016, Zhou et al. 2021, Dastogeer et al. 2022). Several ecological theories have been tested, including the Anna Karenina Principle (Zaneveld et al. 2017, Arnault et al. 2023) and the diversity-invasibility hypothesis (Jousset et al. 2011, van Elsas et al. 2012). The Anna Karenina Principle (AKP) predicts that stress, whether biotic or abiotic, induces dysbiosis, defined as a transient loss of the host control over its microbiota (Arnault et al. 2023). This loss alters microbiota composition and function and can be both the cause and consequence of disease symptoms. According to AKP, dysbiosis alters the processes of microbiota assembly and manifests as increased stochasticity in microbiota assembly, which influences sample dispersion (i.e. the degree of dissimilarity observed between microbial samples) (Arnault et al. 2023). It is also expected to affect microbial α-diversity and load. Several studies have suggested that the microbial load remains under host control until pathogenic species invade host tissues and that successful pathogen colonization is associated with a higher microbial load, either in the natural microbiota (Guo et al. 2020, Karasov et al. 2020) or in a synthetic microbial community (Wolinska et al. 2021). According to the diversity-invasibility hypothesis, increased microbial diversity limits pathogen invasion (Jousset et al. 2011, van Elsas et al. 2012) through at least four mechanisms namely sampling effect, insurance effect, complementarity effect, and synergistic effects (Hooper et al. 2005, Saleem et al. 2019). However, both the Anna Karenina Principle and the diversity-invasibility relationship were recently challenged (Williams et al. 2024). There is still a longstanding debate regarding the relationship between the microbiota and host health. In this study, we aimed to advance this field by investigating the relationship between the plant microbiota and a major disease caused by a pathogenic oomycete species.

Oomycetes cause some of the most devastating crop diseases (Derevnina et al. 2016). Among them, *Plasmopara viticola*, the causal agent of grapevine downy mildew was introduced to Europe in the mid-nineteenth century and devastated European vineyards due to the high susceptibility of *Vitis vinifera* (Fontaine et al. 2021). This foliar disease is now reported in most wine-producing regions of the world (Bois et al. 2017, Fontaine et al. 2021) and has a significant economic impact (Taylor et al. 2019). Several microorganisms have been identified as potential biocontrol agents through experiments conducted under controlled conditions. These potential biocontrol agents include several bacteria of the *Bacillus* genus (Zhang et al. 2017, Bruisson et al. 2019), as well as fungi such as *Acremonium byssoides* (Burruano et al. 2016), *Alternaria* spp. (Musetti et al. 2006), *Epicoccum nigrum* (Kortekamp 1997), *Fusarium* spp. (Ghule et al. 2018), and *Trichoderma* spp. (Perazzolli et al. 2008, Lazazzara et al. 2021). A recent study also discovered *Simplicillium lanosoniveum*, a hyperparasite specific to *P. viticola*, through isolation from grapevine lesions (Shen et al. 2022). In addition, Fournier et al. (2025) reported that fungi such as *Buckleyzyma aurantiaca, Bullera alba, Trichoderma virens*, and *Trichoderma hamatum*, and bacteria like *Streptomyces* and *Bacillus* were more abundant in soil and phyllosphere of vineyard plots with historically low downy mildew symptoms. In particular, basidiomycete yeasts were more abundant in the phyllosphere of low-disease plots. Despite these discoveries, only *Bacillus amyloliquefaciens*, is currently registered in France for controlling *P. viticola* (“DGAL/SAS/2022–949″). Consequently, there is considerable room for improvement in biocontrol strategies targeting grapevine downy mildew. It would be particularly valuable to explore the combination of multiple biocontrol agents (Nicot et al. 2012, Xu and Jeger 2020), building on the diversity-invasibility hypothesis (Saleem et al. 2019). Such combinations of microorganisms are expected to improve the efficiency and robustness of biocontrol by providing functional redundancy and complementarity in modes of action (Guetsky et al. 2007, Vega et al. 2009, Panebianco et al. 2015).

This study aimed to improve microbial biocontrol strategies for the pathogenic oomycete *P. viticola* by deepening our understanding of pathogen-microbiota interactions during infection and by testing debated ecological theories linking microbiota diversity to disease development (Williams et al. 2024). We compared microbial community in asymptomatic and symptomatic tissues of naturally infected leaves to identify taxa consistently more abundant in asymptomatic leaf tissues. We also tested three hypotheses derived from ecological theories. First, we hypothesized that (H1) leaf tissues that were asymptomatic at the time of sampling during an epidemic harbor a more diverse microbiota than symptomatic tissues do, which is consistent with the diversity-invasibility relationship (Saleem et al. 2019). Second, we hypothesized that (H2) the microbial load in asymptomatic tissues is lower than that in symptomatic tissues, in line with the dysbiosis concept, which posits that disease is linked to a loss of host control over its microbiota (Karasov et al. 2020, Arnault et al. 2023). Third, we hypothesized that (H3) infection increases stochasticity in microbiota assembly processes and alters sample dispersion, in accordance with the Anna Karenina principle (Zaneveld et al. 2017, Arnault et al. 2023). Our findings are discussed in the context of developing microbial biocontrol of grapevine downy mildew, with the ultimate goal of reducing grape growers’ reliance on chemical pesticides that are harmful to both human and environmental health (Rani et al. 2021, Mwaka et al. 2024).

## Materials and methods

The experimental design and the dataset have been described in detail in the data paper by (Barroso-Bergadà et al. 2023a), in which the authors presented microbial profiles and rarefaction curves. The same dataset was also previously exploited to investigate microbial interaction networks using an explainable machine learning framework, namely Abductive/Inductive Logic Programming (Barroso-Bergadà et al. 2023b). In the present study, we extended these previous works by supplementing the dataset with information on bacterial and fungal load and by providing a more comprehensive analysis of the microbiota. This included comparisons of α-diversity, β-diversity and microbial load between symptomatic and asymptomatic tissues, the detection of disease-related microbial taxa using four methods of differential abundance analysis and TITAN, and the investigation of community assembly processes.

### Experimental design, *P. viticola* quantification and sequencing of fungal and bacterial communities

The acquisition of the dataset is therefore only briefly summarized here, as it has already been described in detail in (Barroso-Bergadà et al. 2023a). A total of 270 grapevine leaves were collected in 2018 from nine vineyard plots across three major French wine-growing regions (Occitanie, Nouvelle-Aquitaine, hereafter referred to as Aquitaine and Champagne) (Supplementary Fig. S1). during the peak of the grapevine downy mildew epidemic. From each leaf, discs were cut from both symptomatic (visibly sporulating and non-necrotic lesions) and asymptomatic areas, and freeze-dried. Total DNA was extracted and sequenced on an Illumina MiSeq platform to characterize fungal (nrDNA ITS gene) and bacterial (16S rRNA gene) communities. Sequence processing involved the DADA2 pipeline (Callahan et al. 2016), taxonomic assignment using UNITE All Eukaryotes v8.3 (Abarenkov et al. 2021) and SILVA v138.1 (Quast et al. 2013) and clustering using the LULU algorithm (Frøslev et al. 2017). The DNA concentration of *P. viticola* was quantified across all samples with quantitative real-time PCR targeting the ITS1. The concentration was then divided by the total DNA concentration, assumed to consist mainly of leaf DNA, to express the result as ng of *P. viticola* DNA per µg of leaf DNA.

### Quantification of microbial load

In the present study, we provided a new layer of information by quantifying microbial load across all samples using digital droplet PCR assays (ddPCR™; Hindson et al. 2011). Microbial load was defined as the absolute quantity of microbial DNA, serving as a proxy for microbial biomass. The ddPCR assays were conducted with the QX200 Droplet Digital PCR System from Bio-Rad at the Genome Transcriptome Facility of Bordeaux (France). The primers and probes were synthesized by Integral DNA Technologies with probes labeled with 5′6-FAM, an Internal ZENTM and 3′IBFQ quenchers.

Fungal DNA was amplified using the universal fungal primer pair ITS86F (5′-GTGAATCATCGAATCTTTGAA-3′) and ITS4 (5′-TCCTCCGCTTATTGATATGC-3′) as recommended by Beeck et al. (2014). In addition to amplifying fungal sequences, these primers also amplify oomycete sequences, including those of *Plasmopara viticola*, as confirmed by in silico BLAST analyses and confirmed through PCR assays performed in our laboratory (data not shown). PCRs were carried out in a final volume of 22 μl using the ddPCR™ EvaGreen Supermix (Bio-Rad, USA) with 11 µl of 2X EvaGreen Supermix, 1.83 μL of each primer at 1.5 μM and 2 μL of DNA template or ultrapure water as the negative control. A DNA extract of *Phaeomoniella chlamydospora*, a pathogenic fungus responsible for Esca disease in grapevine (González-Domínguez et al. 2020), was used as a positive control.

Bacterial DNA was amplified using the universal bacterial primer pair F_Bact 1369 (5′-CGGTGAATACGTTCCCGG-3′), R_Prok1492 (5′-TACGGCTACCTTGTTACGACTT-3′) and the P_TM1389F (5′-CTTGTACACACCGCCCGTC-3′) as recommended by Furet et al. (2009). PCRs were conducted in a final volume of 22 μl using the ddPCR™ Supermix for Probes No dUTP (Bio-Rad, USA), with 11 µl of 2X Supermix, 2.2 μL of each primer at 9 µM, 1.22 µL of probe at 9 µM and 2 μL of DNA template or ultrapure water in the negative control. The positive control was the DNA of a mock community composed of 14 bacterial strains (*Streptococcus mitis, Streptococcus oralis, Pseudomonas aeruginosa, Stenotrophomonas maltophilia, Staphylococcus epidermidis, Staphylococcus aureus, Acinetobacter baumannii, Klebsiella pneumoniae, Proteus mirabilis, Serratia marcescens, Lactobacillus, Escherichia coli* (ATCC 25922)*, Enterobacter cloacae, and Enterococcus faecalis* (ATCC 29212)).

For both groups, 20 µL of mixture containing the sample was partitioned into droplets with a QX200 Droplet Generator and then transferred to 96-well PCR plates. PCRs were performed in a Bio-Rad C1000 (Bio-Rad, USA) instrument with the following parameters: [95 °C × 5 min; 40 cycles of 95 °C × 30 s, 55 °C × 1 min, and 72 °C × 30 s; 4 °C × 5 min, 90 °C × 5 min] for fungi; and [95 °C × 5 min; 40 cycles of 95 °C × 30 s, 60 °C × 1 min; and 98 °C × 10 min] for bacteria. The QX200 droplet reader analyzed each droplet individually to detect the fluorescence signal. The number of copies of the target sequence per microliter of extracted DNA was calculated from the number of positive droplets (out of an average of ∼20k droplets per sample). The estimated load was then obtained by multiplying the obtained concentration (expressed as the number of copies/µl) by the mix volume sample/buffer (x11) and after adjusting for the 1/100 dilution of the DNA extract. The number of copies was then divided by the total DNA concentration, assumed to consist mainly of leaf DNA, to express it as number of copies per µg of leaf DNA.

### Statistical analysis

All the statistical analyses were performed with R v4.2.3 (R Core Team 2023). Microbial community analyses were performed using the R packages *phyloseq* v1.48.0 (McMurdie and Holmes 2013) and *speedyseq* v0.5.3.9018 (McLaren 2020), and all figures were generated using the *ggplot2* v3 package. 5.1 (Wickham 2016), *cowplot* v1.1.3 (Wilke 2024), *ggh4x* v0.2.8 (van den Brand 2024), ggsignif v0.6.4 (Ahlmann-Eltze and Patil 2021), *patchwork* v1.2.0 (Pedersen 2024), *microViz* v0.10.8 (Barnett et al. 2021), and *ggtext* v0.1.2 (Wilke and Wiernik 2022).

#### Data preparation

Microbial community analyses were either based on the sample × ASV raw count matrices available from Barroso-Bergadà et al. (2023a), or on matrices transformed to account for compositional effects. The compositional effects were accounted for by transforming the raw sequence counts using the centered log-ratio (CLR) transformation (Gloor et al. 2017). Prior to the CLR transformation, we applied a Bayesian multiplicative treatment of zeros using the *cmultRepl* function of the *zComposition* package v1.5.0.3 (Palarea-Albaladejo and Martín-Fernández 2015). This function converts zero counts, which would lead to errors in the log ratios, into estimates close to zero, assuming that these zeros are due to undersampling rather than absence. It also drops rows (ASVs) or columns (samples) with more than 80% zeros or missing data.

To construct phylogenetic trees of ASVs, we first performed multiple sequence alignment using the *AlignSeqs* function of the *DECIPHER* v2.26.0 package (Wright 2016). Next, the phylogenetic distance matrix was calculated via maximum likelihood using the *dist.ml* of the package *phangorn* v2.11.1 (Schliep 2011, Schliep et al. 2016), and the tree was built using the Neighbor-Joining method (*NJ* option of the *phangorn* package). Finally, we evaluated the phylogenetic tree’s likelihood in relation to the alignment and chosen a model using the *pml* function of the *phangorn* package.

#### Comparison of *P. viticola* abundance between asymptomatic and symptomatic leaf tissues

First, we verified that leaf tissues classified as symptomatic based on visual observations contained a higher DNA amount of *P. viticola* than tissues classified as asymptomatic based on qPCR data (available from Barroso-Bergadà et al. (2023a), by using a pairwise Wilcoxon test. The alternative hypothesis that the DNA amount of *P. viticola* is higher in asymptomatic tissues was tested by using the alternative argument = “greater” in the *pairwise.wilcox.test* function of the package *stats* v4.2.3 (R Core Team 2023).

#### Analysis of factors driving variation in leaf microbiota composition

Principal Component Analysis (PCA) was applied to the sample × ASV CLR-transformed matrix to visualize variation in microbial community composition, using the *microViz* package. We identified key factors influencing community composition using variance partitioning and Redundancy Analysis (RDA) implemented using the *varpart* and *rda* functions of the *vegan* package v2.6.4 (Oksanen et al. 2024), respectively. Nine explanatory factors were used in both analyses. For variance partitioning, we categorized the data into three groups: Disease, Geography and Variety. The ‘Disease’ category represented infection by *P. viticola* and included two variables: the *P. viticola* DNA concentration estimated by qPCR (in ng/µl) and visual assessments of downy mildew symptoms (asymptomatic or symptomatic leaf samples). The ‘Variety’ category comprised a single variable, representing the grapevine variety. This variable had seven modalities, corresponding to the grape varieties (Chasan, Chardonnay, Gamay, Merlot, Cabernet Franc, Meunier, Pinot Noir) included in the experiment (Barroso-Bergadà et al. 2023a). The ‘Geography’ category represented the sample location and consisted of six variables. The first variable was the vine leaf number from which the leaf discs were sampled. This variable was used to take into account the pairing between symptomatic and asymptomatic tissue samples collected from the same leaf. The five other variables were the Principal Coordinates of the Neighbourhood Matrix (PCNM; Borcard and Legendre 2002), which represents the spatial distribution of the nine vineyard plots included in the experiment (Barroso-Bergadà et al. 2023a). The PCNMs were generated by (1) calculating a Euclidean distance matrix between all samples using their spatial coordinates (longitude and latitude) with the *distance* function of the *vegan* package and (2) applying the *pcnm* function of the *vegan* package to perform Principal Coordinates Analysis (PCoA) on the truncated matrix, which provided the eigenvectors associated with positive eigenvalues—the PCNMs—that we used as spatial predictors in the RDA. The PCNMs captured both the higher proximity of samples collected from the same plot (plot effect) and the higher proximity of samples collected from the same wine-producing region (region effect). For both variance partitioning and RDA, the sample x ASV CLR-transformed matrix was used as the response variable. Nonnumeric explanatory variables were treated as dummy variables, and all variables were standardized using the *scale* function (package *base* v4.2.3 (R Core Team 2023)). For the RDA, an automatic stepwise selection of explanatory variables, both forward and backward, was performed using the *ordistep* function of the *vegan* package. Finally, an RDA was performed with all selected explanatory variables included as constraints. Permutation tests were performed using the *anova.cca* function of the *vegan* package to assess the significance of the fitted models and to evaluate the marginal effects of the constraints.

#### Identification of microbial taxa specific to asymptomatic and symptomatic leaf samples

To determine whether asymptomatic and symptomatic samples exhibited higher abundances of specific microbial taxa, we used a set of four Differential Abundance Analysis (DAA) methods: ANCOM-BC2 (Lin and Peddada 2024), Maaslin2 (Mallick et al. 2021), LinDA (Zhou et al. 2022) and ZicoSeq (Yang and Chen 2022). We selected these methods recommended in recent methodological studies for the following reasons: they were specifically developed for microbiota analysis by explicitly accounting for zero inflation and compositional effects; they allow the specification of random and covariate effects (Nearing et al. 2022, Yang and Chen 2022, 2023, Regueira-Iglesias et al. 2023). This set of four DAAs was used to compare ASV abundances between asymptomatic and symptomatic samples while accounting for microbial community variation among plots. For all four methods, the plot and leaf number from which the symptomatic and asymptomatic samples were taken were included as random factors. The parameters were set to defaults except for the minimum prevalence threshold, which was set to 10%, and the adjusted p value for an ASV to be considered differentially abundant, which was set to 0.05. All analyses were performed using the sample × ASV raw count matrix. They were performed using the *ANCOMBC* v2.0.3 (Lin and Peddada 2020, Lin et al. 2022), *Maaslin2* v1.12.0 (Mallick et al. 2021), *GUniFrac* v1.8 (Chen et al. 2023), and *MicrobiomeStat* v1.2 (Zhang et al. 2024) packages. For each ASV identified as differentially abundant by at least one of the four DAA methods, we calculated two scores, as described in Fournier et al. (2025): (1) the number of methods that identified this ASV as differentially abundant, ranging from 1 to 4, and (2) the average association coefficient across the methods. To calculate the average association coefficient, the coefficients provided by each DAA method were standardized between 0 and 1 when the ASV was more abundant in asymptomatic samples and between 0 and -1 when the ASV was more abundant in symptomatic samples before calculating the average coefficient across the methods.

In addition, we identified the microbial taxa whose relative abundance covaries with the total DNA concentration of *P. viticola* in leaf samples, as determined by qPCR values, using the Threshold Indicator Taxa ANalysis (TITAN) method of the *TITAN2* package v2.4.3 (Baker et al. 2023). For this analysis, we used the sample x ASV raw count matrix, keeping only ASVs with more than 100 reads in total and present in at least 3 samples, as required by TITAN. The method identified ASVs whose abundance increased as the *P. viticola* DNA concentration decreased and ASVs whose abundance increased as the *P. viticola* DNA concentration increased. These ASVs are hereafter referred to as indicators of low and high *P. viticola* DNA concentrations in leaves. To evaluate the strength of the relationship, we used the standardized Indicator Value (IndVal) score defined by Dufrêne and Legendre (1997) expressed as a *z* score.

#### Testing Hypothesis H1: is microbiota α-diversity higher in leaf tissues that were asymptomatic at the time of sampling during the epidemic than in disease lesion?

To assess whether the α-diversity of the leaf microbial communities was higher in asymptomatic leaf tissues than in symptomatic leaf tissues, we calculated three diversity indices. These indices are part of the Hill number framework (Chao et al. 2014), which includes a parameter *q* that determines the sensitivity of the indices to the relative abundance of ASVs. This framework gives less weight to rare ASVs as *q* increases. The Hill number corresponding to *q* = 0 represents the richness of ASVs, where each ASV counts for 1 regardless of its relative abundance. The Hill number corresponding to *q* = 1 is the exponential of Shannon’s entropy index (Shannon 1948), where the weight of each ASV is proportional to its relative abundance. The Hill number corresponding to *q* = 2 is the inverse of Simpson’s concentration index (Simpson 1949), which disproportionately favors abundant ASVs and is particularly relevant for metabarcoding data, as rare ASVs often correspond to artifacts, and their inclusion can lead to erroneous ecological conclusions (Taberlet et al. 2018). The three indices were calculated from the sample × ASV raw count matrix using the *ChaoRichness, ChaoShannon*, and *ChaoSimpson* functions in the *iNEXT* v3.0.1 package (Chao et al. 2014, Hsieh and Chao 2024). Microbiota diversity was compared between asymptomatic and symptomatic leaf samples using linear mixed-effects models. We built six models, each corresponding to a combination of the microbial kingdom (bacteria or fungi) and the α-diversity index (*q*=1, 2 or 3). These models included visual assessments of downy mildew symptoms (asymptomatic *vs*. symptomatic leaf samples) as a fixed effect and leaf number as a random effect to consider the pairing of symptomatic and asymptomatic samples taken from the same leaf. Additional fixed effects, such as grapevine variety, plot, and region, were also included to control for potential confounding factors affecting microbial diversity. Graphical checks for homoscedasticity and normality of residuals were performed using the packages *performance* v0.12.0 (Lüdecke et al. 2021) and *DHARMa* v0.4.6 (Hartig and Lohse 2022). Model construction and evaluation were conducted using the packages *lmerTest* v3.1.3 (Kuznetsova et al. 2017) and *car* v3.1.2 (Fox and Weisberg 2018). When explanatory factors with more than two levels were significant, post hoc tests were conducted using the *emmeans* function of the *emmeans* v1.10.1 package (Lenth et al. 2024) to estimate marginal means for each factor level. To adjust for multiple comparisons, we applied the Bonferroni method using the *pairs* function of the *graphics* v4.2.3 package. Finally, the means were ranked in descending order, and groupings were identified using the *cld* function of the *lsmeans* v2.30.0 package (Lenth 2016) to highlight significant differences between factor levels.

#### Testing Hypothesis H2: do fungal and bacterial loads increase in disease lesions caused by the oomycete *P. viticola*, suggesting a loss of plant control over its microbiota?

Linear mixed-effects models were also used to compare microbial loads measured by digital droplet PCR between asymptomatic and symptomatic leaf samples. We built two models, one for each microbial kingdom (bacteria or fungi). The models included visual assessments of downy mildew symptoms (asymptomatic *vs*. symptomatic leaf samples) as a fixed effect and leaf as a random effect to consider the pairing of symptomatic and asymptomatic samples taken from the same leaf. Additional fixed effects, such as grapevine variety, plot, and region. Model evaluation was performed as described above.

#### Testing Hypothesis H3: does infection increase stochasticity in microbiota assembly processes and alter sample dispersion, in accordance with the Anna Karenina principle?

We quantified the ecological processes driving microbial community assembly in both asymptomatic and symptomatic tissues, using the β-nearest-taxon index (βNTI) and the normalized stochasticity ratio (NST). The βNTI (Stegen et al. 2012) quantifies phylogenetic turnover between site pairs by comparing observed mean nearest taxon distances (βMNTDs) to null expectations, thereby distinguishing stochastic from deterministic community assembly processes. The NST (Ning et al. 2019) estimates the relative influence of stochasticity by comparing observed community similarity to that expected under null models, yielding a normalized value indicative of stochastic or deterministic structuring.

We calculated their values for every sample following the procedures described by Barnett et al. (2020) and Ning et al. (2019), and we performed all the statistical tests according to their outlined methods. For NST calculation, we used the function *pNST*, which estimates NST on the basis of phylogenetic beta diversity (Guo et al. 2018, Ning et al. 2019), which we estimated using the β-mean-nearest-taxon distance (βMNTD). This method has been shown to perform better in stochasticity estimation than NST based on taxonomic dissimilarity indices in several cases (Ning et al. 2020).

According to Stegen et al. (2013), β-NTI values between –2 and 2 indicate a dominance of stochastic processes, whereas |β-NTI| >2 reveals the dominance of deterministic processes. NST values classify community assembly as more stochastic (>50%) or more deterministic (<50%) (Ning et al. 2019). To compare βNTI values between symptomatic and asymptomatic leaf samples, we performed a Kruskal-Wallis test. NST values were compared between the two groups using bootstrap resampling (*nst.boot* function from the *NST* package).

To evaluate the effect of disease symptoms on sample dispersion, we first calculated the weighted UniFrac distance as a β-diversity index using the *distance* function in the *phyloseq* package. We then quantified the sample dispersion by calculating the distance of each sample to its group centroid using the *betadisper* function from the *vegan* package (comparing symptomatic and asymptomatic tissues). Differences in dispersion between groups were tested for statistical significance using the *permutest* function, also from the same package.

## Results

Our results complement and extend previous analyses of the same dataset, which included microbial community profiles (Barroso-Bergadà et al. 2023a), and illustrated a new network learning method using the fungal dataset (Barroso-Bergadà et al. 2023b).

### qPCR data confirm minimal infection of asymptomatic tissues by *P. viticola*

As expected, the DNA amount of *P. viticola* was significantly lower in asymptomatic leaf tissues than in symptomatic leaf tissues (paired Wilcoxon test, p < 0.001, n = 446) (Supplementary Fig. S2). In asymptomatic tissues, the *P. viticola* DNA amount was very low with a median value of 0.07 ng per µg of leaf DNA, and there was minimal variability among samples (Supplementary Fig. S2). In disease lesions, however, the *P. viticola* DNA amount was significantly higher, with a median value of 193 ng/µg and there was considerable variability among samples (Supplementary Fig. S2). A few samples showed similarly low levels to those found in asymptomatic tissues. Conversely, a small number of asymptomatic samples showed measurable levels of *P. viticola* DNA, in some cases comparable to those observed in symptomatic tissues. These results support the relevance of classifying samples into two categories (asymptomatic *vs*. symptomatic) based on visual observations of symptoms while also highlighting both a gradient of *P. viticola* DNA amount within symptomatic tissues—which may reflect different stages of infection—and the occasional detection of high levels of pathogenic DNA in visually asymptomatic samples.

### Geography, and to a lesser extent grapevine variety, have more influence on microbiota composition than infection by downy mildew

**Figure 1.**
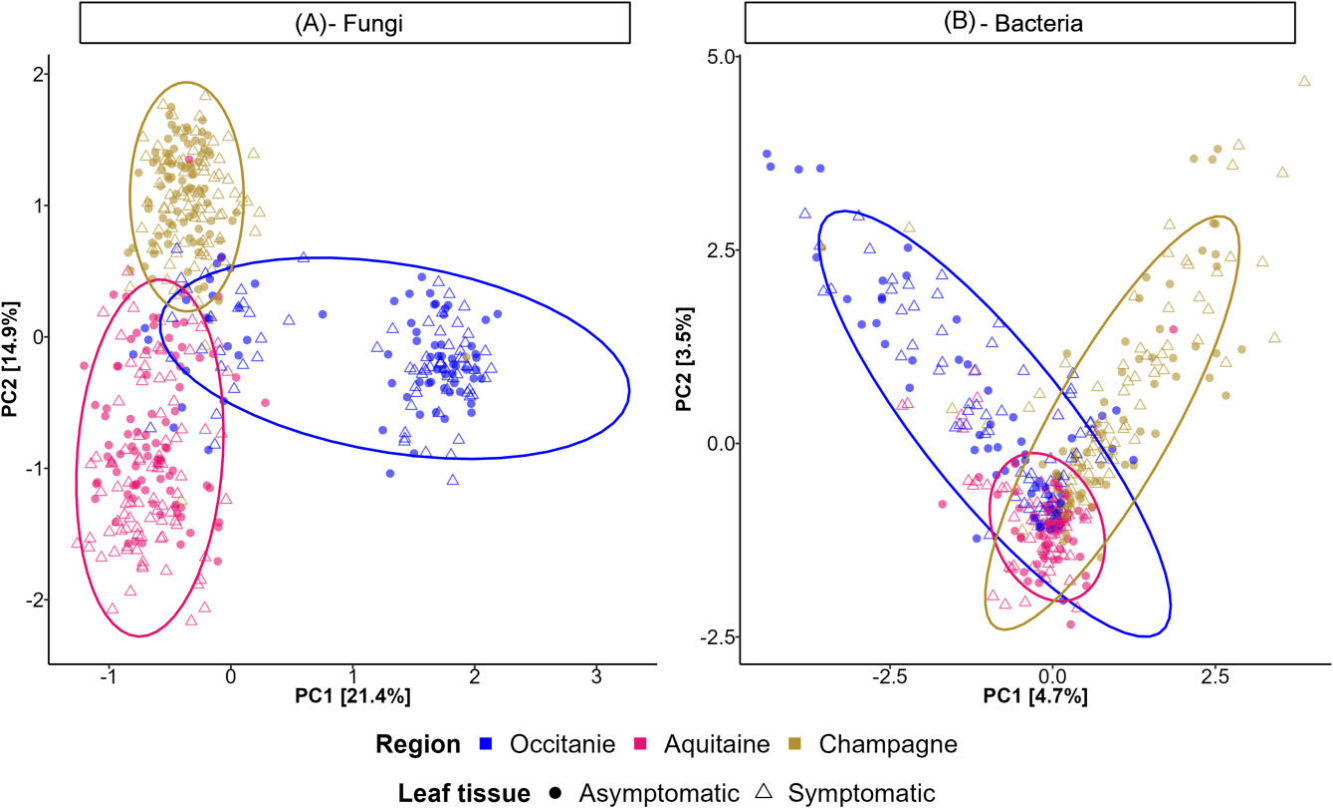
Variation in the microbial community composition of grapevine leaves sampled during a downy mildew epidemic across three French wine-growing regions. **Compositional** dissimilarities between **(A)** fungal and **(B)** bacterial communities of grapevine leaves, represented by a Principal Component Analysis (PCA). Samples collected from the same wine-growing region are represented with the same color and are enclosed within an ellipse. Symbols indicate leaf tissue status: circles for asymptomatic leaves and triangles for downy mildew-symptomatic ones.

Fungal communities of grapevine leaves were spatially structured, with marked differences in composition among the three wine-producing regions (Figs. 1A and 3A and Supplementary Fig. S3A). Geography was the main driver of fungal community variation, accounting for 36.82% of the variance, followed by grapevine variety (6.16%) and infection by downy mildew (0.59%) (Supplementary Fig. S4A). According to the redundancy analysis (RDA), all three factors had a significant effect (Supplementary Table S1). The bacterial communities were less spatially structured than the fungal communities were but still exhibited regional variation across the three wine-producing regions (Fig. 1B; Supplementary Fig. S3B). Geography was also the main driver of variation in bacterial community composition, explaining 15.06% of the variance, followed by grapevine variety (3.31%) and infection by downy mildew (0.51%) (Supplementary Fig. S4B). However, according to the redundancy analysis (RDA), only geography had a significant effect on the bacterial communities (Supplementary Table S1). Importantly, however, the grapevine variety was not uniformly distributed across regions: each variety was sampled in a single plot, except for Chardonnay (which was sampled in Occitanie and Champagne) and Merlot (which was sampled in two plots in Aquitaine). Consequently, the effect of grapevine variety may be partially confounded by plot or regional effects in both fungal and bacterial community analyses.

**Figure 2.**
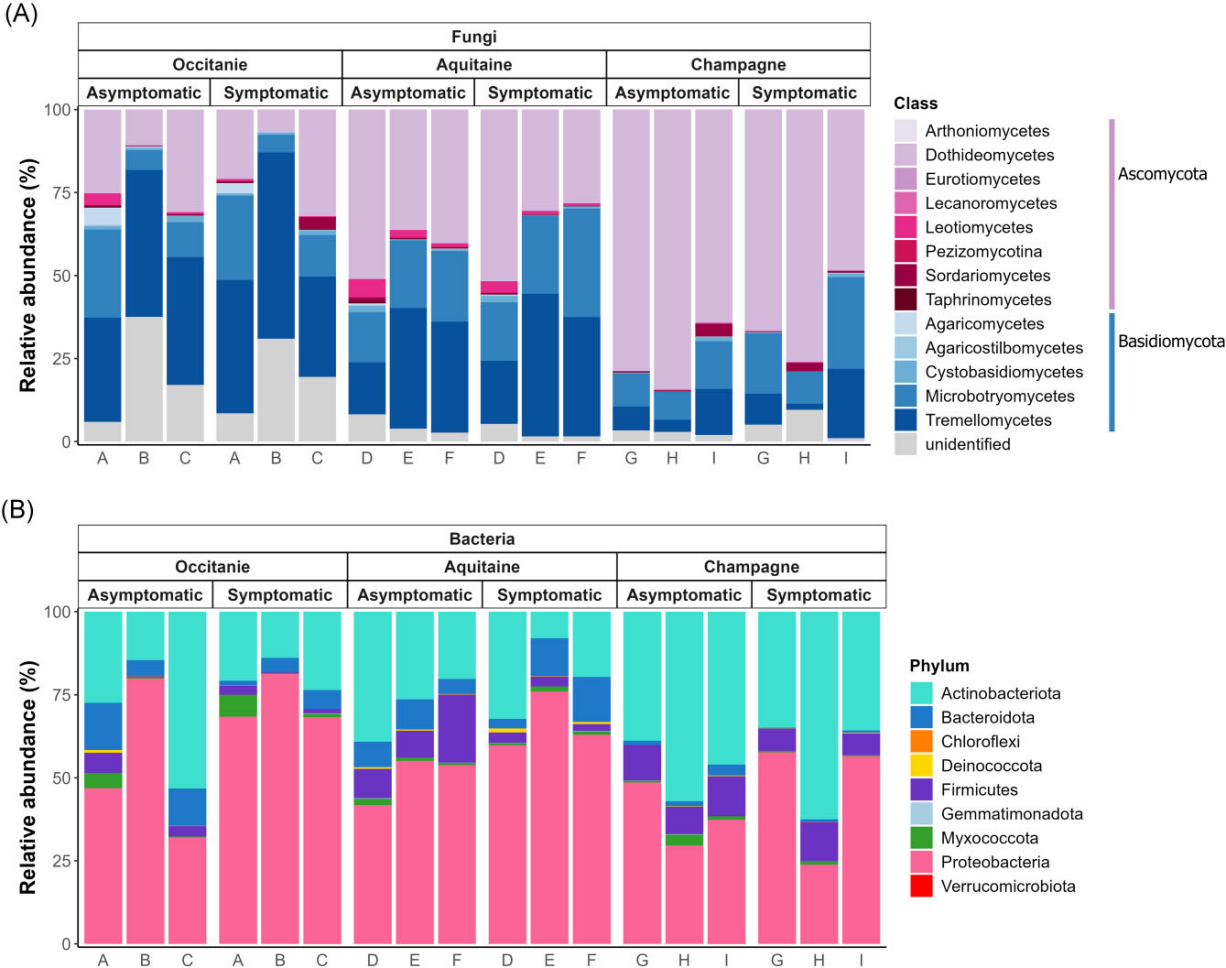
Microbial community profiles of grapevine leaves according to geographic region and leaf tissue status (asymptomatic *vs*. downy mildew-symptomatic). **(A)** Fungal and **(B)** bacterial community profiles in asymptomatic and downy mildew-symptomatic grapevine leaf discs collected during the peak of the downy mildew epidemic across nine plots (A–I) in three French wine-growing regions: Occitanie, Aquitaine and Champagne (Fig. 1). The relative abundances of fungal classes and bacterial phyla are averaged over the 30 samples collected for each condition. In the fungal community profiles, the Ascomycota classes are represented by a pink gradient, whereas the Basidiomycota classes are represented by a blue gradient.

Fungal community profiles varied between southern (Occitanie) and northern (Champagne) regions of France (Fig. 2A), with a marked shift from higher proportions of Basidiomycetes—particularly the Tremellomycetes class—in southern France to increased proportions of Ascomycetes, especially Dothideomycetes, in northern France. The most abundant fungal species were generally shared across regions (Table 1), but their total relative abundance varied with the South to North gradient: they represented 40.8% of the species in Occitanie, 60.6% in Aquitaine and 86.8% in Champagne. In addition, their relative abundances varied between regions. Among the top 10 most abundant fungal species present in the 3 regions were the basidiomycete yeasts *Filobasidium chernovii, Filobasidium oeirense, Vishniacozyma victoriae*, and *Sporobolomyces roseus*, as well as the ascomycetes *Cladosporium delicatulum* and *Mycosphaerella tassiana*. The latter increased markedly in abundance from South to North (7.07% in Occitanie, 9.88% in Aquitaine, and 48.10% in Champagne), contributing significantly to the higher proportion of Ascomycetes in the northern region. Other species, such as *Filobasidium wieringae, Alternaria metachromatica*, and *Stemphylium solani*, were among the top 10 in two of the three regions. Other species, such as *Phebia rufa* and *Vishniacozyma carnescens* in Occitanie, *Itersonilia pannonica* and *Udeniomyces pyricola* in Aquitaine or *Dioszegia hungarica* and *Bulleromyces albus* in Champagne, were present in only one region. Despite similarities in the core bacterial genera across regions, the relative abundance of the dominant taxa varied geographically. The genera *Sphingomonas, Frigobacterium, Pantoea, Curtobacterium*, and *Methylobacterium* were consistently among the top 10 most abundant genera across all three regions (Table 2). Notably, *Sphingomonas* decreased in abundance from South to North (36.95% in Occitanie, 24.20% in Aquitaine, and 6.46% in Champagne), reflecting a regional gradient similar to that observed for fungal taxa.

**Table 1.** Most abundant fungal species of grapevine leaves in three French wine-growing regions.

French region								
Occitanie (Plots A, B, and C)			Aquitaine (Plots D, E, and F)			Champagne (Plots G, H, and I)		
Fungal species	Relative abundance (%) ± SE	Prevalence (%)	Fungal species	Relative abundance (%) ± SE	Prevalence (%)	Fungal species	Relative abundance (%) ± SE	Prevalence (%)
***Sporobolomyces roseus*** (Basidiomycota)	13.37 ± 0.99^a^	96.71	***Sporobolomyces roseus*** (Basidiomycota)	19.38 ± 0.96^b^	97.13	***Mycosphaerella tassiana*** (Ascomycota)	48.1 ± 1.11^c^	100
***Mycosphaerella tassiana*** (Ascomycota)	7.07 ± 0.47^a^	95.39	***Filobasidium chernovii*** (Basidiomycota)	12.4 ± 1.09^c^	97.13	***Sporobolomyces roseus*** (Basidiomycota)	14.23 ± 0.84^a^	100
***Cladosporium delicatulum*** (Ascomycota)	5.4 ± 0.41^a^	84.87	***Mycosphaerella tassiana*** (Ascomycota)	9.88 ± 0.64^b^	99.43	***Cladosporium delicatulum*** (Ascomycota)	13.63 ± 0.47^b^	98.3
***Filobasidium chernovii*** (Basidiomycota)	3.87 ± 0.42^b^	88.16	*Filobasidium wieringae* (Basidiomycota)	7.31 ± 0.81	87.36	***Vishniacozyma victoriae*** (Basidiomycota)	3.5 ± 0.48^c^	97.16
*Filobasidium wieringae* (Basidiomycota)	3.22 ± 0.68	48.03	***Cladosporium delicatulum*** (Ascomycota)	5.34 ± 0.42^a^	83.91	*Alternaria metachromatica* (Ascomycota)	3.45 ± 0.26	86.36
*Vishniacozyma carnescens* (Basidiomycota)	2.33 ± 0.31	95.39	***Vishniacozyma victoriae*** (Basidiomycota)	1.56 ± 0.21^b^	89.66	***Filobasidium chernovii*** (Basidiomycota)	1.59 ± 0.27^a^	66.48
***Filobasidium oeirense*** (Basidiomycota)	2.13 ± 0.44^b^	82.24	*Itersonilia pannonica* (Basidiomycota)	1.42 ± 0.20	71.84	*Stemphylium solani* (Ascomycota)	0.96 ± 0.18	91.48
*Stemphylium solani* (Ascomycota)	1.27 ± 0.85	94.08	***Filobasidium oeirense*** (Basidiomycota)	1.15 ± 0.30^a^	59.2	***Filobasidium oeirense*** (Basidiomycota)	0.5 ± 0.08^a^	76.7
*Phlebia rufa* (Basidiomycota)	1.1 ± 0.93	2.63	*Alternaria metachromatica* (Ascomycota)	1.12 ± 0.112	76.44	*Dioszegia hungarica* (Basidiomycota)	0.46 ± 0.04	89.77
***Vishniacozyma victoriae*** (Basidiomycota)	1.03 ± 0.24^a^	72.37	*Udeniomyces pyricola* (Basidiomycota)	1.02 ± 0.154	85.63	*Bulleromyces albus* (Basidiomycota)	0.42 ± 0.06	93.75
**Total**	40.89		**Total**	60.58		**Total**	86.84	

The table shows the top ten most abundant fungal species, independently of the leaf tissue, in Occitanie, Aquitaine and Champagne. Species in bold are among the top ten most abundant species in all three regions, while those underlined are among the top ten most abundant species in 2 out of 3 regions. Relative abundance (%) ± SE indicates the proportion of sequences assigned to the species relative to the total number of sequences in the dataset, with the standard error (SE). Prevalence (%) indicates the percentage of samples where the species is present with at least one sequence. For each species among the top ten most abundant in all three regions, different superscript letters indicate significant differences in relative abundance according to Dunnett’s test (α = 0.05).

**Table 2.** Most abundant bacterial genera on grapevine leaves in three French wine-growing regions.

French region								
Occitanie (Plots A, B, and C)			Aquitaine (Plots D, E, and F)			Champagne (Plots G, H, and I)		
Bacterial genus	Relative abundance (%) ± SE	Prevalence (%)	Bacterial genus	Relative abundance (%) ± SE	Prevalence (%)	Bacterial genus	Relative abundance (%) ± SE	Prevalence (%)
***Sphingomonas*** (Proteobacteria)	36.95 ± 2.79^b^	73.77	***Sphingomonas*** (Proteobacteria)	24.2 ± 1.96^b^	80.16	***Frigoribacterium*** (Actinobacteriota)	11.01 ± 1.22^b^	36.62
***Frigoribacterium*** (Actinobacteriota)	10.71 ± 1.44^b^	49.18	*Hymenobacter* (Bacteroidota)	6.76 ± 1.12	53.17	***Pantoea*** (Proteobacteria)	9.97 ± 0.97^b^	25.35
*Massilia* (Proteobacteria)	9.56 ± 1.44	50.82	***Methylobacterium*** (Proteobacteria)	5.63 ± 0.81^b^	78.57	***Curtobacterium*** (Actinobacteriota)	7.09 ± 1.01^b^	21.83
*Pseudomonas* (Proteobacteria)	6.65 ± 1.47	40.16	***Pantoea*** (Proteobacteria)	4.15 ± 0.77^a^	4.76	***Sphingomonas*** (Proteobacteria)	6.46 ± 1.14^a^	67.61
***Pantoea*** (Proteobacteria)	5.9 ± 1.45^ab^	15.57	*Massilia* (Proteobacteria)	3.66 ± 0.66	37.3	*Bacillus* (Firmicutes)	4.37 ± 0.82	67.61
*Hymenobacter* (Bacteroidota)	4.98 ± 1.02	53.28	*Pseudomonas* (Proteobacteria)	2.59 ± 0.40	36.51	***Methylobacterium*** (Proteobacteria)	2.67 ± 0.44^a^	51.41
***Methylobacterium*** (Proteobacteria)	4.23 ± 0.60^a^	45.9	*Streptococcus* (Firmicutes)	2.34 ± 0.61	23.02	*Streptomyces* (Actinobacteriota)	2.55 ± 0.63	33.1
***Curtobacterium*** (Actinobacteriota)	2.14 ± 1.13^a^	7.38	*Friedmanniella* (Actinobacteriota)	2.12 ± 0.53	24.6	*Rhodococcus* (Actinobacteriota)	2.48 ± 0.57	35.92
*Kineococcus* (Actinobacteriota)	1.11 ± 0.59	14.75	***Frigoribacterium*** (Actinobacteriota)	2.02 ± 0.33^a^	13.49	*Nocardioides* (Actinobacteriota)	2.28 ± 0.31	80.99
*Arthrobacter* (Actinobacteriota)	0.86 ± 0.29	18.85	***Curtobacterium*** (Actinobacteriota)	1.67 ± 0.40^a^	3.97	*Pseudarthrobacter* (Actinobacteriota)	2.09 ± 0.59	50
**Total**	83.09		**Total**	55.14		**Total**	50.97	

The table shows the top ten most abundant bacterial genera, independently of the leaf tissue, in Occitanie, Aquitaine and Champagne. Genera in bold are among the top ten most abundant genera in all three regions, while those underlined are among the top ten most abundant genera in 2 out of 3 regions. Relative abundance (%) ± SE indicates the proportion of sequences assigned to the genus relative to the total number of sequences in the dataset, with the standard error (SE). Prevalence (%) indicates the percentage of samples where the genus is present with at least one sequence. For each genus among the top ten most abundant in all three regions, different superscript letters indicate significant differences in relative abundance according to Dunnett’s test (α = 0.05).

### Some fungi, but not bacteria, are consistently more abundant in asymptomatic leaf tissues across wine-producing regions

**Figure 3.**
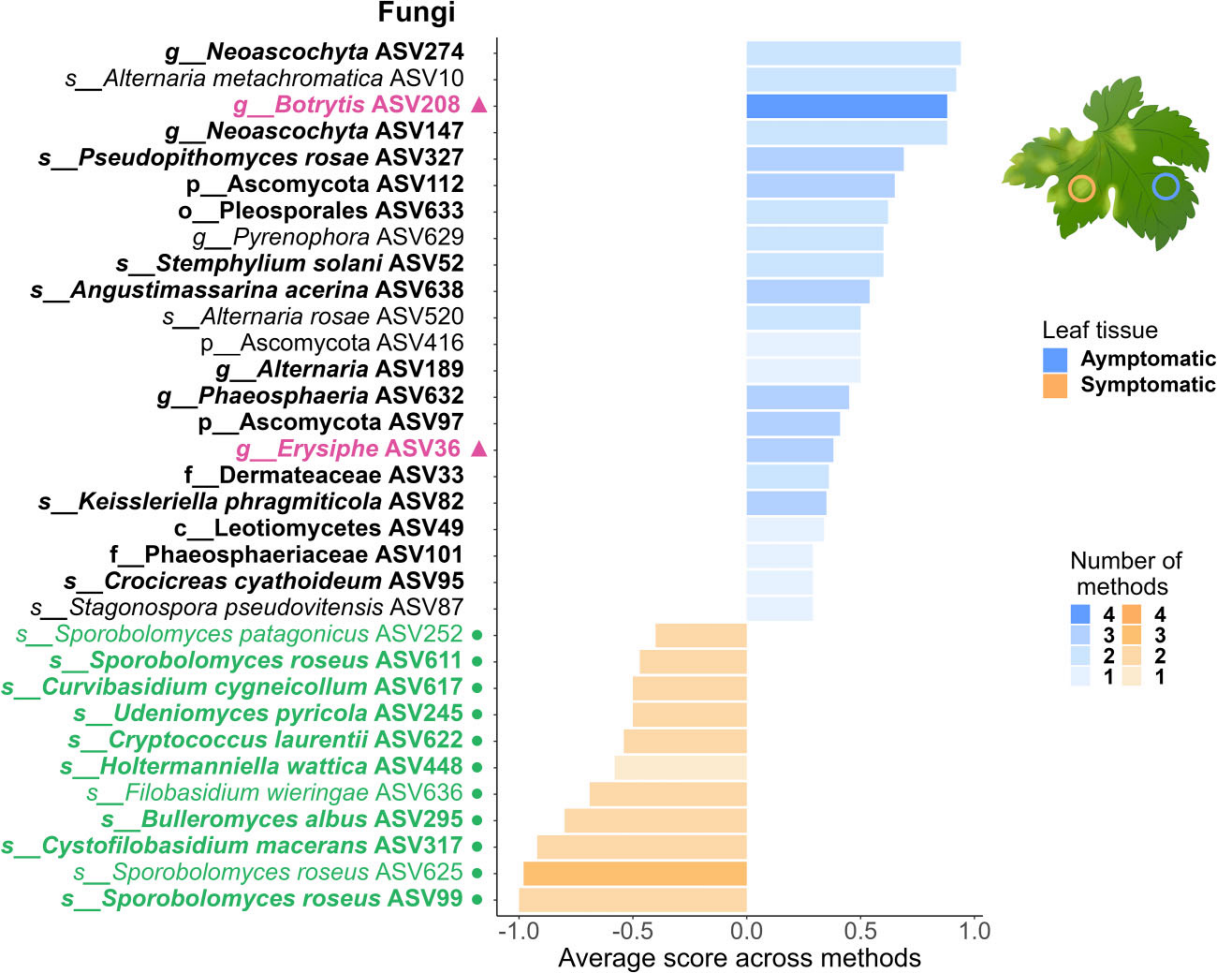
Fungal ASVs that vary in abundance between asymptomatic and downy mildew-symptomatic grapevine leaf tissue. For each condition (asymptomatic *vs*. symptomatic), we represented the ASVs that were significantly more abundant in that condition according to at least one method of differential abundance analysis (DAA). The four methods used to identify these ASVs are ANCOM-BC2 (Lin and Peddada 2024), MaAslin2 (Mallick et al. 2021), LinDA (Zhou et al. 2022), and ZicoSeq (Yang and Chen 2022). All analyses were conducted at the national level (i.e. using the full dataset combining all three regions). Shades of blue and orange indicate the number of DAA methods that identified the ASV as differentially abundant (ranging from 1 to 4). ASVs belonging to basidiomycete yeasts are highlighted in green and marked with a dot while those known as foliar pathogens of grapevine are highlighted in pink and marked with a triangle. ASVs that do not meet either of these criteria are displayed in black without any specific symbol. ASVs that are also significant in the TITAN analysis (Baker et al. 2023) are shown in bold. The y-axis provides information on the lowest taxonomic level at which each ASV was identified, including its prefix, assignment, and number of ASVs. The prefix abbreviations are *p* for phylum, *c* for class, *o* for order, *f* for family, *g* for genus, and *s* for species.

**Figure 4.**
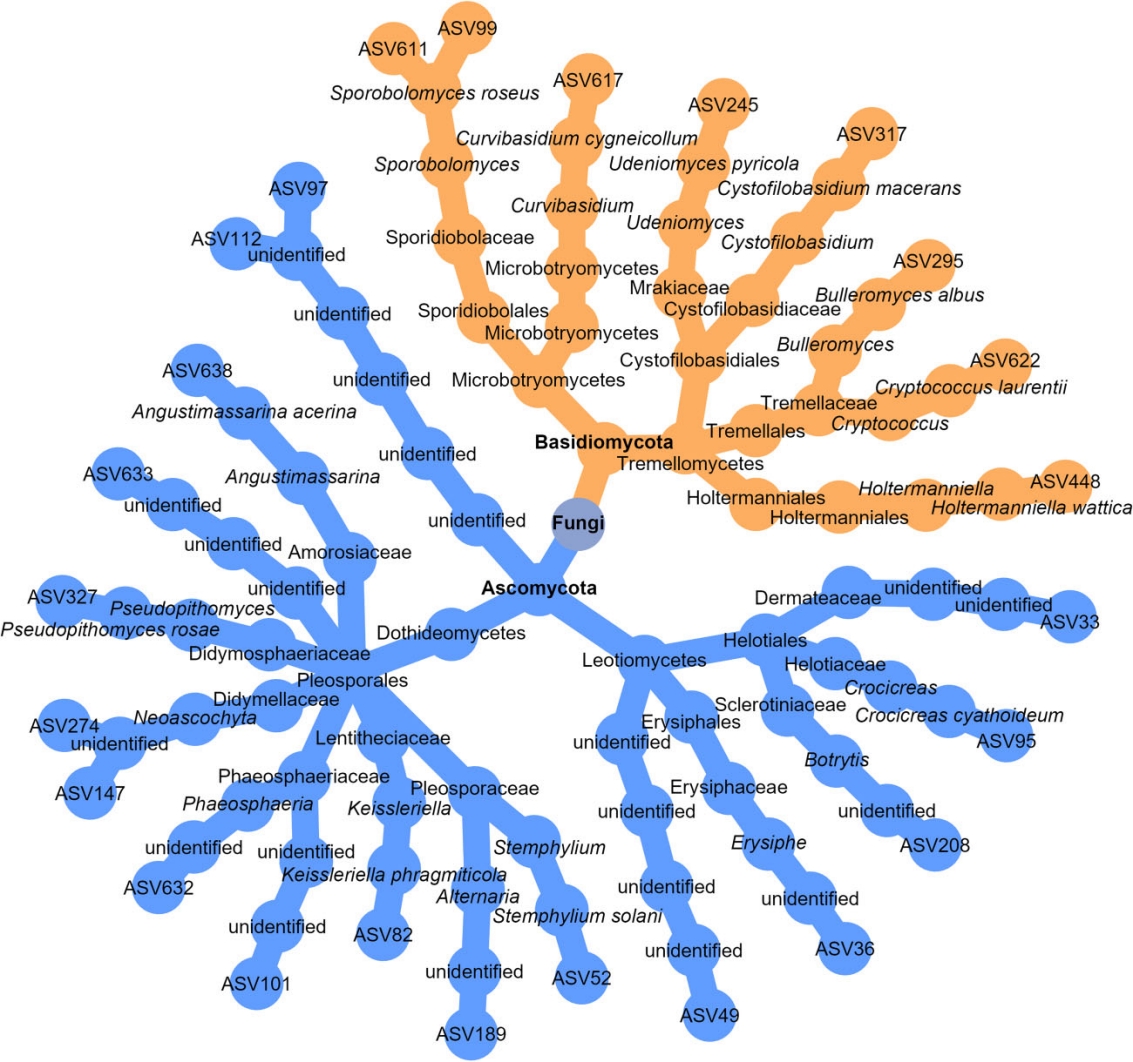
Taxonomic tree of fungal ASVs that vary in abundance according to *P. viticola* DNA concentration and leaf tissue status (asymptomatic *vs*. symptomatic). Orange branches correspond to ASVs that meet both of the following criteria: (i) identified by at least one differential abundance analysis (DAA) method as more abundant in lesions and (ii) with abundance positively correlated with *P. viticola* DNA concentration in tissues (according to TITAN analysis (Baker et al. 2023)). The blue branches correspond to ASVs identified by at least one DAA as more abundant in asymptomatic tissues and negatively correlated with the *P. viticola* DNA concentration. The four DAA methods used were ANCOM-BC2 (Lin and Peddada 2024), MaAslin2 (Mallick et al. 2021), LinDA (Zhou et al. 2022), and ZicoSeq (Yang and Chen 2022). All analyses were conducted at the national level (i.e. using the full dataset combining all three regions). Note that all the ASVs shown in orange belong to the Basidiomycota phylum, whereas those in blue are exclusively from the Ascomycota phylum.

Differential abundance analysis (DAA) conducted at the national level (i.e. using the full dataset combining all three regions) identified 22 fungal ASVs that were significantly more abundant in asymptomatic leaf tissues (Supplementary File S2), whereas TITAN identified 78 fungal ASVs whose abundance was negatively correlated with the DNA concentration of *P. viticola* in leaf tissues (Supplementary File S3). Seventeen fungal ASVs were identified by both DAA and TITAN (Fig. 3), all of which belong to the Ascomycota phylum (Fig. 4). To assess region-specific patterns, DAAs were then performed separately on each regional dataset. These results revealed that both Occitanie and Aquitaine harbored distinct sets of fungal taxa that were more abundant in asymptomatic tissues (Figs. S4 and S5), whereas no such taxa were detected in Champagne (Fig. S6). Interestingly, at the national scale, two fungal ASVs assigned to pathogenic genera, *Erysiphe* (ASV36) and *Botrytis* (ASV208), were significantly more abundant in asymptomatic tissues when considering the full dataset encompassing all three regions (Fig. 3). Similar patterns emerged from the regional analyses: *Botrytis* (ASV208) was more abundant in asymptomatic tissues in both Occitanie and Aquitaine, while *Erysiphe* (ASV36) was more abundant in asymptomatic tissues in Aquitaine (Figs. S5 and S6).

**Figure 5.**
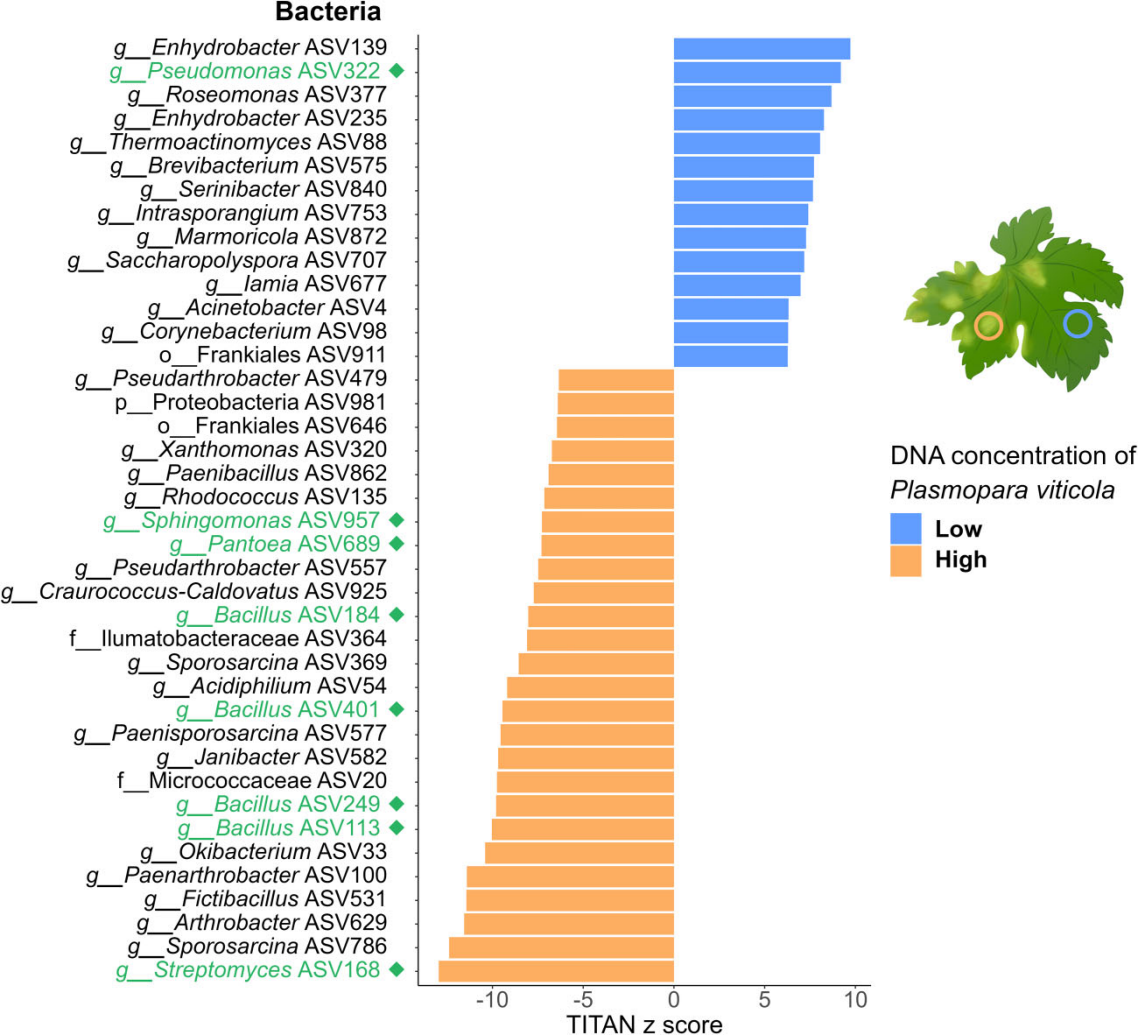
Bacterial taxa whose abundance covaries with the ***P. viticola* DNA concentration** in grapevine leaf tissues. The figure displays ASVs whose abundance is significantly correlated with the *P. viticola* DNA concentration in leaf tissue, as identified by TITAN analysis (Baker et al. 2023). ASVs in blue are negatively correlated with *P. viticola* DNA levels, indicating that they are more abundant when its concentration is low. Conversely, ASVs in orange are positively correlated, being more abundant at high *P. viticola* DNA levels. The analysis was conducted at the national level (i.e. using the full dataset combining all three regions). The y-axis provides information on the lowest taxonomic level at which the ASV was identified, including its prefix, assignment, and number of ASVs. The prefix abbreviations are *p* for phylum, *c* for class, *o* for order, *f* for family, *g* for genus, and *s* for species. The 40 ASVs with the highest z scores are shown in this figure. Members of *Bacillus, Pantoea, Pseudomonas, Sphingomonas* and *Streptomyces* are shown in green followed by a diamond.

In contrast, DAA conducted at the national level did not identify any bacterial ASVs that were significantly more abundant in asymptomatic leaf tissues (Supplementary File S2 and Supplementary Fig. S8). However, TITAN identified 33 bacterial ASVs whose abundance was negatively correlated with the DNA concentration of *P. viticola* in leaf tissues (Supplementary File S3; Fig. 5). When applied separately to each regional dataset, DAA identified three bacterial ASVs that were significantly more abundant in asymptomatic tissues in the Occitanie region: one assigned to the *Pseudokineococcus* genus and two to the *Methylobacterium* genus (Supplementary Fig. S9).

### Basidiomycetous yeasts and Bacillales increase in abundance in disease lesions of *P. viticola*

DAA conducted at the national level revealed that 11 fungal ASVs were significantly more abundant in disease lesions, all of which were basidiomycetous yeasts. These genera included *Bulleromyces albus, Cryptococcus laurentii, Curvibasidium cygneicollum, Cystofilobasidium macerans, Filobasidium wieringae, Holtermanniella wattica, Sporobolomyces patagonicus, Sporobolomyces roseus* (represented by three different ASVs), and *Udeniomyces pyricola* (Fig. 3). Eight of these ASVs were also identified as significant by TITAN (Fig. 3 and Fig. 4). This predominance of basidiomycetous yeasts in symptomatic tissues was consistently observed when analyses were conducted at the regional scale (Supplementary Figs. S6-S7), particularly in Aquitaine, where 8 out of 9 differentially abundant ASVs were assigned to this group. Five of these ASVs were also significant according to TITAN (Supplementary Fig. S6). Notably, *Sporobolomyces roseus* was consistently found to be significantly more abundant in disease lesions, both across all regions and within individual regions, except for Occitanie, where no fungal ASVs were identified as more abundant in disease lesions (Fig. 3 and Supplementary Figs. S5-S7).

DAAs revealed that only a few bacterial ASVs were significantly more abundant in disease lesions when analyses were conducted at the national level (2 ASVs assigned to *Pantoea* and one to *Frigoribacterium*) (Supplementary Fig. S8). Region-specific analyses also revealed differentially abundant bacterial ASVs in Occitanie—*Sphingomonas, Frigoribacterium*, and *Massilia* (2 ASVs)—and in Champagne—*Streptomyces* and *Bacillus* (2 ASVs)—but none were detected in Aquitaine (Supplementary Figs. S9–S10). TITAN identified 43 bacterial ASVs whose abundance was positively correlated with the DNA concentration of *P. viticola* in leaf tissues (Fig. 5; Supplementary File S3). Among the positively correlated ASVs, four were assigned to *Bacillus*, three to *Pseudomonas*, three to *Pantoea*, one to *Sphingomonas*, and one to *Streptomyces* (Supplementary File S3). Fig. 5 shows a subset of the 40 ASVs with the highest z scores, including both positively and negatively correlated ASVs; consequently, not all the ASVs positively associated with *P. viticola* are represented in the figure. The complete list is available in Supplementary File S3.

### The microbial α-diversity and bacterial load both decrease in disease lesions

**Figure 6.**
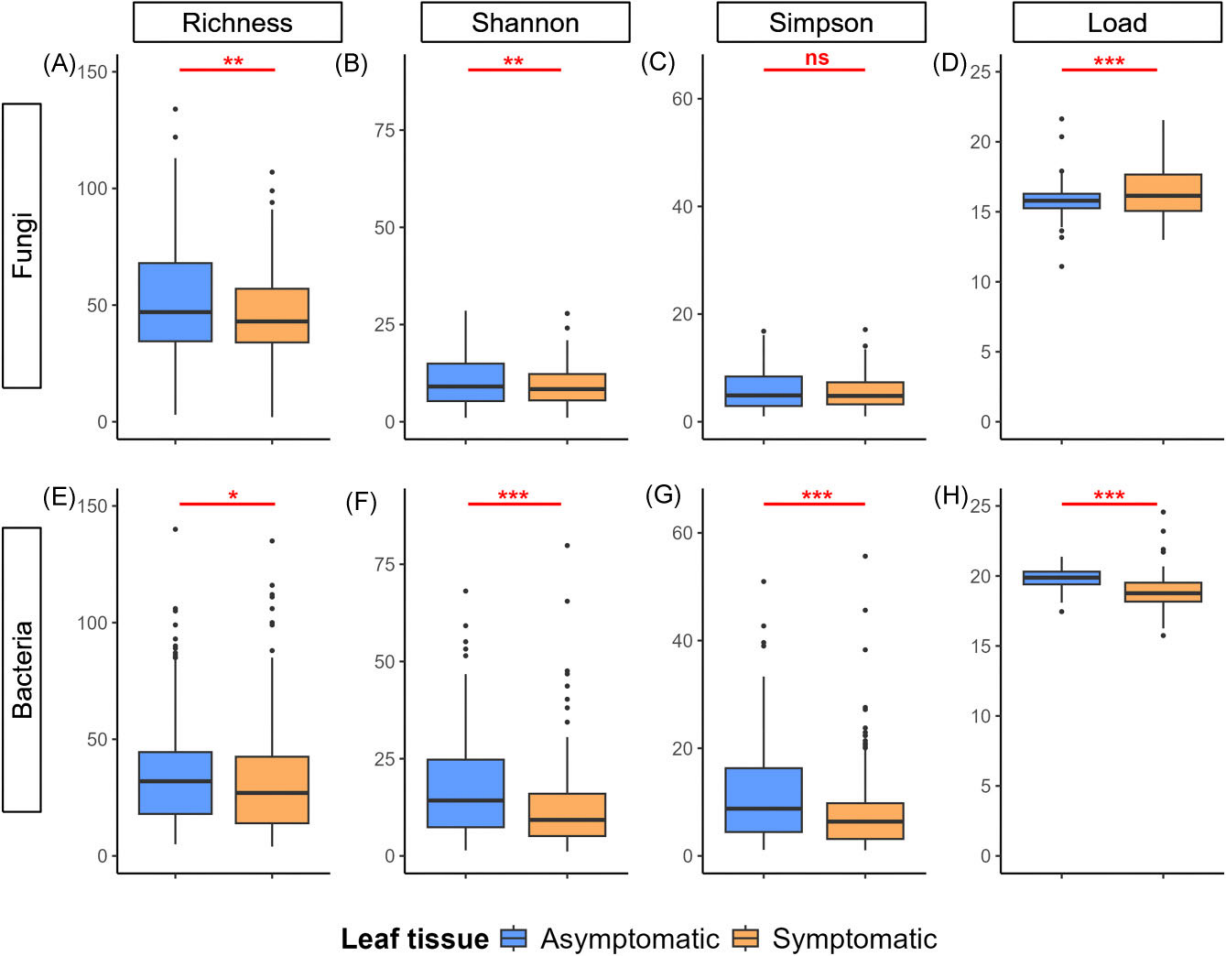
Variation in microbial α-diversity and load between asymptomatic and downy mildew-symptomatic grapevine leaf tissue. The figure shows the diversity and load of (A-D) fungal and (E-H) bacterial communities in grapevine leaves collected at the peak of the downy mildew epidemic. The metrics presented, from left to right, are Richness (panels A for fungi and E for bacteria), the observed total number of ASVs (Hill number q = 0); Shannon (B and F), the exponential of the Shannon entropy index (Hill number q = 1); Simpson (C and G), the inverse of the Simpson concentration index (Hill number q = 2); and Load (D and H), the microbial load (expressed as the number of copies/ng of plant DNA and estimated using ddPCR). The statistical significance of the linear mixed effects models is indicated as follows: ns (not significant), * (*P*<0.05), ** (*P*<0.01), *** (*P*<0.001). The detailed statistical results are presented in Table S2.

The bacterial and fungal community α-diversities were significantly higher in leaf tissues that were asymptomatic at the time of sampling during the downy mildew epidemic (Fig. 6A-C for fungi and Fig. 6E-G for bacteria; Table S2), in accordance with hypothesis H1. However, in the case of fungal communities, the difference was not significant when the inverse Simpson’s index was used to estimate α-diversity (Fig. 6C), suggesting that the variation in diversity between asymptomatic tissues and disease lesions was due primarily to rare fungal ASVs.

The bacterial load was significantly lower in symptomatic tissues according to ddPCR analysis (Fig. 6H; Table S2), which contradicts our initial expectation (H2). In contrast, the fungal load was significantly higher in symptomatic tissues than in asymptomatic tissues (Fig. 6D; Table S2). This increase is likely caused by the increase in *P. viticola* DNA amount in disease lesions, as the primers used for ddPCR can amplify both fungi and *P. viticola*. According to the results of the qPCR analysis, the *P. viticola* DNA amount increased sharply in symptomatic tissues, with a multiplicative ratio of 45. In comparison, the overall increase in ITS copy number/µg of leaf DNA measured by ddPCR was more moderate, with a multiplicative ratio of 3.2. This discrepancy could reflect a decrease in fungal load in symptomatic tissues—similar to what was observed for bacteria.

### Selective processes in microbiota assembly increase in disease lesions

**Figure 7.**
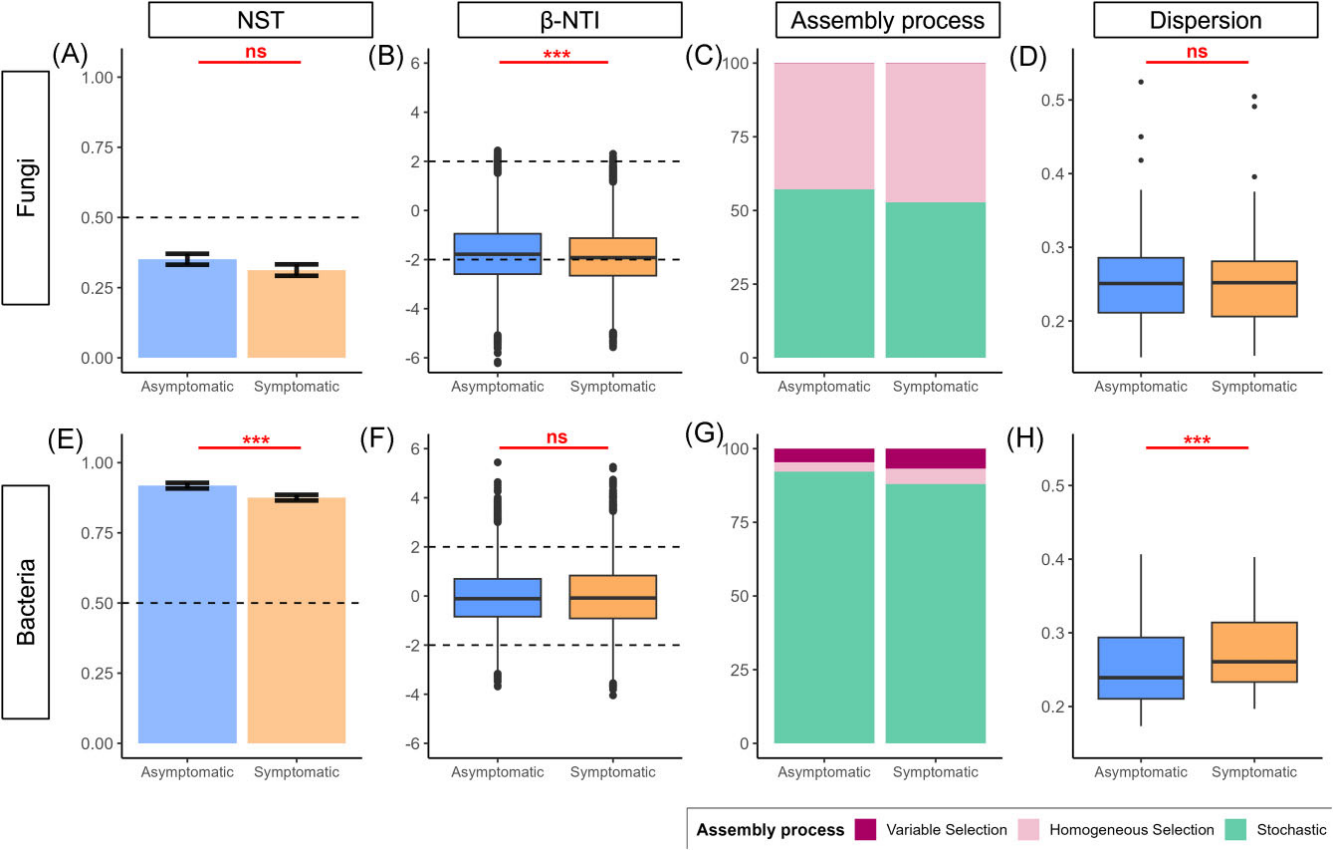
Contributions of deterministic and stochastic processes to the assembly of microbial communities in asymptomatic and downy mildew-symptomatic grapevine leaf tissue. The figure shows the assembly processes (calculated with two indices, NST and βNTI) and the sample dispersion for the (A–D) fungal and (E–H) bacterial communities. NST (panels A for fungi and E for bacteria) quantifies the proportion of stochastic *vs*. deterministic processes in microbial community assembly. A value of 50% is used as a cutoff (indicated by a dashed line in the figure), with <50% indicating a more deterministic assembly and >50% indicating a more stochastic assembly. βNTI (B and F) measures phylogenetic turnover between pairs of samples. βNTI < -2 indicates a significant effect of homogeneous selection, βNTI > 2 indicates a significant effect of variable selection, and -2 < βNTI < 2 indicates a dominance of stochastic processes. The dashed lines represent the significance thresholds for βNTI. The assembly process (C and G) indicates the percentage of sample pairs assigned to each process. Finally, the dispersion (D and H) represents the distance to the centroid of each sample based on weighted UniFrac distances.

Analyses of the β-NTI and NST indices revealed contrasting community assembly processes between fungi (Fig. 7A-C) and bacteria (Fig. 7E-G). Indeed, the bacterial community assemblage was predominantly governed by stochastic processes (Fig. 7E-G), while deterministic and stochastic processes contributed almost equally to the assembly of fungal communities (Fig. 7A-C). According to the NST values (<50%), fungal communities even tended to be shaped more by deterministic processes (Fig. 7A).

Deterministic processes in fungi were solely due to homogeneous selection (Fig. 7C), whereas in bacteria, these processes were more balanced between homogeneous selection and variable selection (Fig. 7G). Contrary to hypothesis (H3), stochasticity did not increase in disease lesions. Instead, selective processes increased slightly in disease lesions for both fungal communities (according to the β-NTI index) (Fig. 7B) and bacterial communities (according to the NST index) (Fig. 7E).

The bacterial communities associated with disease lesions were significantly more dissimilar from each other (i.e. more dispersed) than were those associated with asymptomatic tissues (Fig. 7H). However, this pattern did not hold for the fungal communities (Fig. 7D).

## Discussion

In the present study, we applied state-of-the-art methods in microbial community ecology to the metabarcoding datasets provided by Barroso-Bergadà et al. (2023a) and complemented these datasets with additional quantitative data. We compared the diversity, composition, and assembly processes of microbial communities between asymptomatic leaf tissues and downy mildew lesions across three French wine-growing regions. This detailed, theory-driven analysis allowed us to propose a novel scenario of interactions involving fungi, bacteria, and the pathogenic oomycete *P. viticola*, the causal agent of grapevine downy mildew.

### A new scenario of interactions between the grapevine leaf microbiota and *P. viticola*

Our analyses revealed that the microbial communities associated with asymptomatic tissues and disease lesions harbored very similar taxonomic compositions for both fungi and bacteria, despite the significant increase in the DNA amount of the pathogen in disease lesions and the visible symptoms caused to the leaf tissues. Overall, we detected only subtle changes in the composition of the leaf microbiota triggered by infection. These changes were driven by selective processes, according to our analyses of community assembly processes.

In the case of fungi, leaf tissue infection by *P. viticola* locally selected for basidiomycetous yeasts, such as *Sporobolomyces patagonicus, Sporobolomyces roseus, Cryptococcus laurentii*, and *Udeniomyces pyricola*. These yeasts increased in abundance upon infection across all wine-growing regions considered. In the case of bacteria, leaf tissue infection by *P. viticola* locally selected for certain groups of bacteria, such as Bacillales and Streptomycetales. In both kingdoms, these compositional shifts were accompanied by reduced bacterial load (but not fungal load) and α-diversity, and a minor increase in community assembly determinism—suggesting that lesions act as environmental filters.

Surprisingly, the leaf tissues that were asymptomatic at the time of sampling during the epidemic were not consistently associated with the fungi or bacteria known for their biocontrol properties. Instead, they were associated with other airborne pathogens of grapevines. The *Erysiphe* and *Botrytis* genera, which comprise species responsible for powdery mildew and gray mold, respectively, increased in abundance in asymptomatic tissues. These results suggest that leaf infection by *P. viticola* locally excludes other pathogens and selects for specific microbial taxa, some of which may have biocontrol activities, such as basidiomycetous yeasts and *Bacillus* species.

### Fungi and bacteria with known biocontrol properties are selected for in disease lesions of grapevine downy mildew

According to our analyses, basidiomycetous yeasts were consistently selected for within disease lesions. We used several methods to identify the fungal taxa that were enriched in disease lesions compared with asymptomatic tissues or whose abundance is positively correlated with the *P. viticola* DNA concentration. All the fungal taxa that met the two criteria were basidiomycetous yeasts belonging to the classes Microbotryomycetes and Tremellomycetes, including *Sporobolomyces roseus, Cryptococcus laurentii, Curvibasidium cygneicollum, Cystofilobasidium macerans, Bulleromyces albus, Holtermanniella wattica*, and *Udeniomyces pyricola. S. roseus* was previously identified as a potential competitor of *P. viticola* based on interaction network analysis using the same dataset (Barroso-Bergadà et al. 2023b). Surprisingly, however, our results show that *S. roseus* is enriched in diseased lesions, suggesting that its ecological role may be more complex than simple antagonism. Basidiomycetous yeasts are well known for their biocontrol activity against postharvest diseases (Liu et al. 2013, Spadaro and Droby 2016, Freimoser et al. 2019). They reduce the development of pathogens through various mechanisms, including competition for nutrients and space; secretion of toxins, enzymes, and volatile organic compounds; direct parasitism; and indirect mechanisms such as resistance induction (Liu et al. 2013, Spadaro and Droby 2016, Freimoser et al. 2019). Fournier et al. (2025) found basidiomycete yeasts to be significantly more abundant in the phyllosphere of young grapevine leaves from plots with historically low downy mildew pressure. This suggests that they may contribute to early-season protection against *P. viticola* infection. Thus, these yeasts are naturally present in the phyllosphere early in the growing season (Fournier et al. 2025) and appear to increase in abundance within disease lesions according to our results.

We also identified several bacterial taxa whose abundance increased with that of *P. viticola*. These strains belong to the genera *Streptomyces, Bacillus, Pantoea, Pseudomonas*, and *Sphingomonas*, most of which contain strains known for their biocontrol activity against grapevine downy mildew (Compant et al. 2013, El-Sharkawy et al. 2018, Bruisson et al. 2019). In particular, *Bacillus* species are well known for their biocontrol activities, through direct antibiosis, competition for niches and nutrients and induction of host systemic resistance (Compant et al. 2013). They produce various bioactive compounds, such as surfactin, iturin, and fengycin, which display strong suppressive effects on a wide range of pathogens (Li et al. 2019). The application of live strains of *Streptomyces* or *Bacillus* bacteria, or their extracts, has been shown to reduce disease severity (El-Sharkawy et al. 2018, Li et al. 2019 ) by inhibiting and lysing zoospores (Abdalla et al. 2011), disrupting zoospores motility (Islam et al. 2016, Raveau et al. 2024)), and damaging sporangia and sporangiophores (Liang et al. 2016). *Pantoea agglomerans* and *Sphingomonas zeae* are also considered potential antagonists of *P. viticola* according to *in vitro* confrontation tests (Bruisson et al. 2019).

To our knowledge, our study is the only one to specifically investigate the microbiota in grapevine downy mildew lesions using metabarcoding, and to directly compare microbial communities between symptomatic and asymptomatic tissues of the same leaf. Other studies have compared grapevine microbiota composition under conditions of low versus high downy mildew abundance, but their experimental designs differed substantially. The comparisons were carried out either between resistant and susceptible grapevine cultivars (Wicaksono et al. 2023, Duret et al. 2025), between treated and untreated plants during a downy mildew epidemic (Duret et al. 2025), between plants from plots with historically low or high incidence and severity of the disease (Fournier et al. 2025), or between different levels of disease severity (Perazzolli et al. 2014). These studies mainly focused on the bacterial microbiota (Supplementary Tables S3) and identified several genera that were significantly more abundant under each of the compared conditions (Supplementary Tables S3 and S4). For example, several bacterial genera were reported as more abundant under low downy mildew abundance, including *Paracoccus* and *Altererythrobacter*, which were negatively correlated with disease severity (Perazzolli et al. 2014) and enriched in resistant cultivars (Wicaksono et al. 2023, Duret et al. 2025). Similarly, *Rosemonas* was negatively correlated with downy mildew severity (Perazzolli et al. 2014) and enriched in plots with historically low levels of disease (Fournier et al. 2025). These findings are consistent with our results, as we also found these taxa to be more abundant in asymptomatic tissues. In addition, Duret et al., (2025) identified bacterial taxa with potential biocontrol activity under both conditions, supporting the hypothesis that high downy mildew abundance could also serve as a reservoir of biocontrol agents. Overall, further comparisons among studies remain challenging due to differences in plant organs analyzed and experimental designs (Supplementary Tables S3 and S4).

### The plant host does not lose control of its microbiota in disease lesions, in contrast to ecological theories

Moreover, in contrast with our initial hypothesis H2, our results suggest that the plant host does not lose control of its microbiota in diseased tissues, at least for fungi. Fungal and bacterial biomass did not increase in disease lesions but rather decreased, and the processes driving community assembly became more deterministic. Homogeneous selection of fungal communities slightly increased in disease lesions compared to asymptomatic tissues, suggesting that infection altered fungal communities in a similar way among all vine plants across the three geographic regions. These deterministic and convergent changes in fungal communities in disease lesions could result from environmental filtering imposed by changes in leaf structure, physiology and chemistry. Indeed, leaf infection by *P. viticola* manifests as yellow and oily spots on the leaves, which evolve into necrotic tissues (Gessler et al. 2011) and trigger significant changes in the concentration and spatial distribution of several micro- and macronutrients (Cesco et al. 2020). Alternatively, the deterministic and convergent changes in fungal communities in disease lesions could result from the active selection of specific microorganisms by the plant in response to infection-induced stress (Teixeira et al. 2019). This pattern is consistent with the “cry-for-help” hypothesis, whereby plants actively recruit beneficial microbes—such as biocontrol-active fungi—in response to biotic stress to fight or resist stress (Raaijmakers and Mazzola 2016, Rizaludin et al. 2021).

Overall, our analysis of microbiota assembly processes did not support the Anna Karenina Principle (AKP). Fungal communities in disease lesions are not more dissimilar from each other (i.e. they are not more dispersed) than are communities in asymptomatic tissues, a pattern that is usually used to support the AKP (Ahmed et al. 2019, Bonthond et al. 2023, Arnault et al. 2023). Moreover, changes in fungal communities between asymptomatic tissues and disease lesions did not correspond to any of the theoretical scenarios proposed by (Arnault et al. 2023), as we did not detect an increase in stochastic processes in disease lesions (AKP pattern) or a shift between predominantly heterogeneous and homogeneous selection (anti-AKP pattern). The bacterial communities were driven mostly by stochastic processes, regardless of the leaf tissue condition (symptomatic or asymptomatic), suggesting that the bacterial communities were loosely controlled by the plant under both conditions.

### Perspectives

One of the most promising findings from our study, which shifts our initial approach, is that symptomatic tissues serve as a valuable reservoir for protective fungal and bacterial microorganisms. The discovery of a significant number of biocontrol agents in downy mildew lesions, from both fungal and bacterial kingdoms, opens exciting new avenues for exploring their complementarity in biological control. With the growing interest in bacterial-fungal interactions, designing SynComs (Synthetic Microbial Communities) that integrate both fungal and bacterial candidates hold the potential to uncover powerful synergies between these organisms, which could significantly advance biocontrol strategies. To fully realize this potential, enhancing the taxonomic assignment of key bacterial ASVs is essential. Multiomics approaches, combining metagenomics, metatranscriptomics and metabolomics, offer promising solutions capable of dramatically improving the precision of bacterial identification and potentially revealing the precise identities of *Bacillus, Streptomyces*, and *Sphingomonas* found in lesions. In addition, these methods would enable us to elucidate the metabolites produced and exchanged in the lesion (Crandall et al. 2020) to develop a mechanistic scenario of interactions between fungi, bacteria and the pathogenic oomycete *P. viticola*.

## Supplementary Material

fiaf111_Supplemental_Files

## Data Availability

Absolute abundance data for fungi and bacteria are available from Recherche Data Gouv under the DOI [https://doi.org/10.57745/H2GJQA]. Other data were published in Barroso-Bergadà et al. (2023a) and are available on Recherche Data Gouv under the DOI [https://doi.org/10.15454/2YDSBL]. All R scripts developed in this study are available on Recherche Data Gouv under the DOI [https://doi.org/10.57745/3DVHRH].
