# Supplementary material for: A new scenario of pathogen-microbiota interactions involving the oomycete Plasmopara viticola
Source: FEMS Microbiol Ecol. 2025 Nov 6;101(12):fiaf111. doi: 10.1093/femsec/fiaf111 (PMC12616103; doi:10.1093/femsec/fiaf111)
Supplement: fiaf111_Supplemental_Files [file fiaf111_supplemental_files.zip › Fournier_et_al_BCMICROBIOME_SupplementaryFileS1_22sept25_rev.docx]

# A new scenario of pathogen-microbiota interactions involving the oomycete *Plasmopara viticola*

Paola Fournier^1^, Lucile Pellan^2^, Julie Aubert^3^, Patrice This^4^, Corinne Vacher^1^

(1) INRAE, Bordeaux Sciences Agro, ISVV, SAVE, Villenave-d’Ornon, France

(2) Ecophysiologie et Génomique Fonctionnelle de la Vigne (EGFV), Université de Bordeaux, Bordeaux sciences Agro, INRAE, ISVV, Villenave d’Ornon, France

(3) Université Paris-Saclay, AgroParisTech, INRAE, UMR MIA-Paris-Saclay, Palaiseau, France

(4) UMR AGAP Institut, Univ Montpellier, CIRAD, INRAE, Institut Agro, Montpellier, France

Paola Fournier: paola.fournier@inrae.fr, https://orcid.org/0000-0002-5743-6599

Lucile Pellan: [lucile.pellan@inrae.fr](mailto:lucile.pellan@inrae.fr), https://orcid.org/0009-0004-6271-7063

Julie Aubert: [julie.aubert@inrae.fr](mailto:julie.aubert@inrae.fr), https://orcid.org/[0000-0001-5203-574](callto:0000-0001-5203-5748)8

Patrice This: patrice.this@inrae.fr, https://orcid.org/0000-0002-3024-5813

Corinne Vacher: corinne.vacher@inrae.fr, https://orcid.org/0000-0003-3023-6113

*Corresponding author: Corinne Vacher

## Supplementary File S1

**Table of contents**

[Supplementary figures 3](#_Toc209458699)

[Figure S1 : Experimental design to investigate the interactions between the grapevine leaf microbiota and the oomycete pathogen *Plasmopara viticola* during a downy mildew epidemic. 3](#_Toc209458700)

[Figure S2 : DNA amount of *Plasmopara viticola* in grapevine leaf tissue, depending on the absence or presence of downy mildew symptoms. 4](#_Toc209458701)

[Figure S3 : Variation in microbial community composition across vineyard plots. 5](#_Toc209458702)

[Figure S4 : Factors driving variation in microbial community composition according to variance partitioning. 6](#_Toc209458703)

[Figure S5 : Fungal ASVs in the Occitanie region that vary in abundance between asymptomatic and downy mildew-symptomatic grapevine leaf tissue. 7](#_Toc209458704)

[Figure S6 : Fungal ASVs in the Aquitaine region that vary in abundance between asymptomatic and downy mildew-symptomatic grapevine leaf tissue. 8](#_Toc209458705)

[Figure S7 : Fungal ASVs in the Champagne region that vary in abundance between asymptomatic and downy mildew-symptomatic grapevine leaf tissue. 9](#_Toc209458706)

[Figure S8 : Bacterial ASVs that vary in abundance between asymptomatic and downy mildew-symptomatic grapevine leaf tissue. 10](#_Toc209458707)

[Figure S9 : Bacterial ASVs in the Occitanie region that vary in abundance between asymptomatic and downy mildew-symptomatic grapevine leaf tissue. 11](#_Toc209458708)

[Figure S10 : Bacterial ASVs in the Champagne region that vary in abundance between asymptomatic and downy mildew-symptomatic grapevine leaf tissue. 12](#_Toc209458709)

[Supplementary tables 13](#_Toc209458710)

[Table S1 : Factors driving variation in microbiota composition according to the redundancy analysis. 13](#_Toc209458711)

[Table S2 : Factors driving variation in microbial α-diversity and load. 14](#_Toc209458712)

[Table S3: List of all studies that investigated the relationship between the grapevine bacterial microbiota and downy mildew. 15](#_Toc209458713)

[Table S4: List of all studies that investigated the relationship between the grapevine fungal microbiota and downy mildew. 22](#_Toc209458714)

[**References** 27](#_Toc209458715)

## Supplementary figures


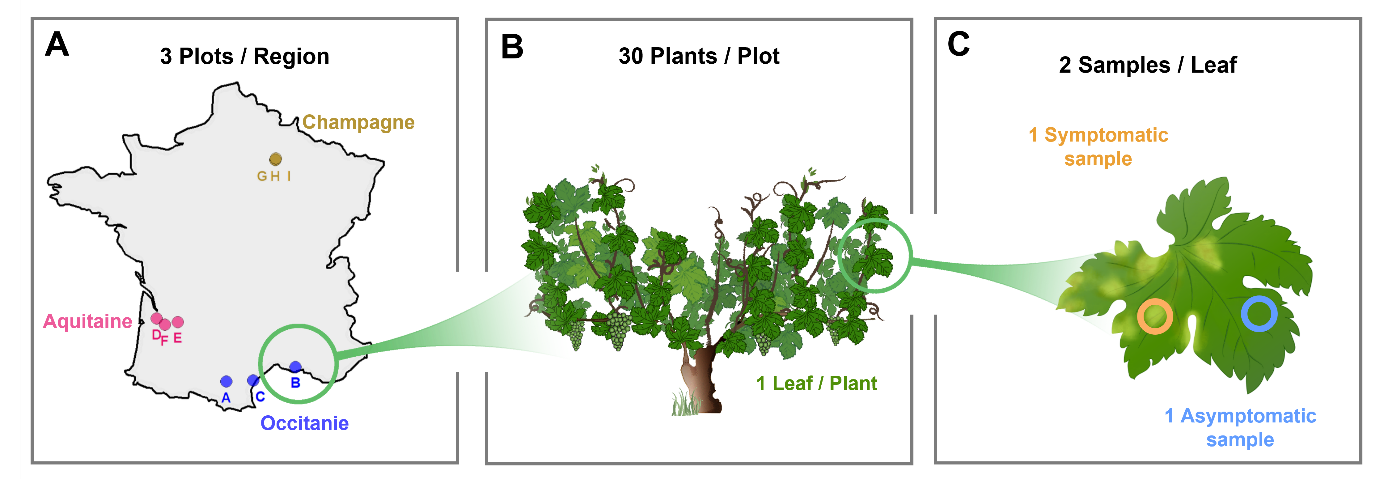


##### Figure S1 : Experimental design to investigate the interactions between the grapevine leaf microbiota and the oomycete pathogen *Plasmopara viticola* during a downy mildew epidemic.

(A) In three viticultural regions of France (Occitanie, Aquitaine, and Champagne), three vineyard plots were sampled. (B) From each plot, 30 leaves harboring downy mildew symptoms were collected from 30 grapevine plants at the peak of the downy mildew epidemic. (C) On each leaf, two paired samples were taken: one from symptomatic tissue (sporulating, nonnecrotic lesions) and one from asymptomatic tissue, resulting in a total of 540 samples (270 paired samples). Each sample consisted of two leaf discs (not shown in the figure for clarity).


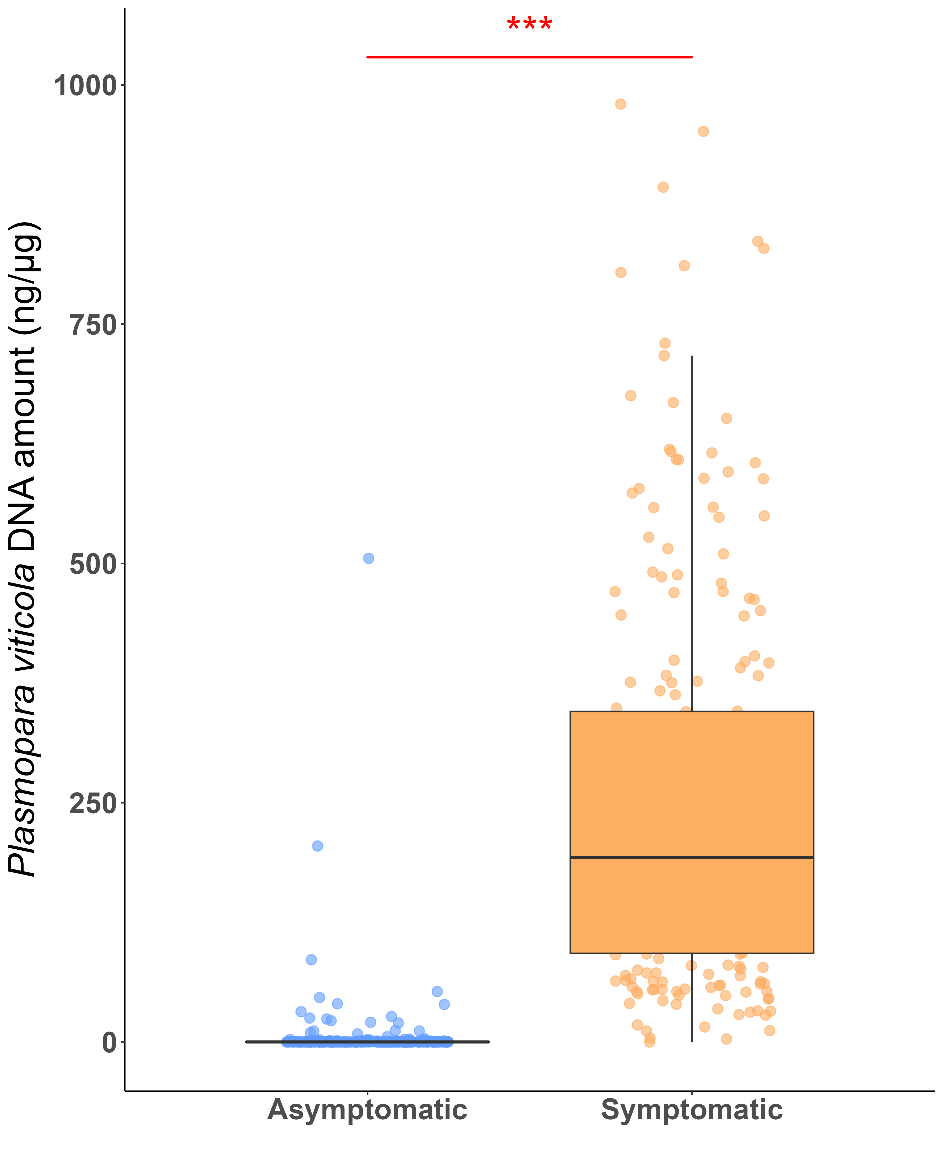


##### Figure S2 : DNA amount of *Plasmopara viticola* in grapevine leaf tissue, depending on the absence or presence of downy mildew symptoms.

The DNA amount of *P. viticola* (in ng/µg of total DNA) was estimated with qPCR. Each point represents a sample, i.e., a disc of asymptomatic (in blue) or diseased (in orange) leaf tissue. The difference in *P. viticola* DNA amount between symptomatic and asymptomatic leaf tissues was assessed using a pairwise Wilcoxon test (*** p < 0.001).


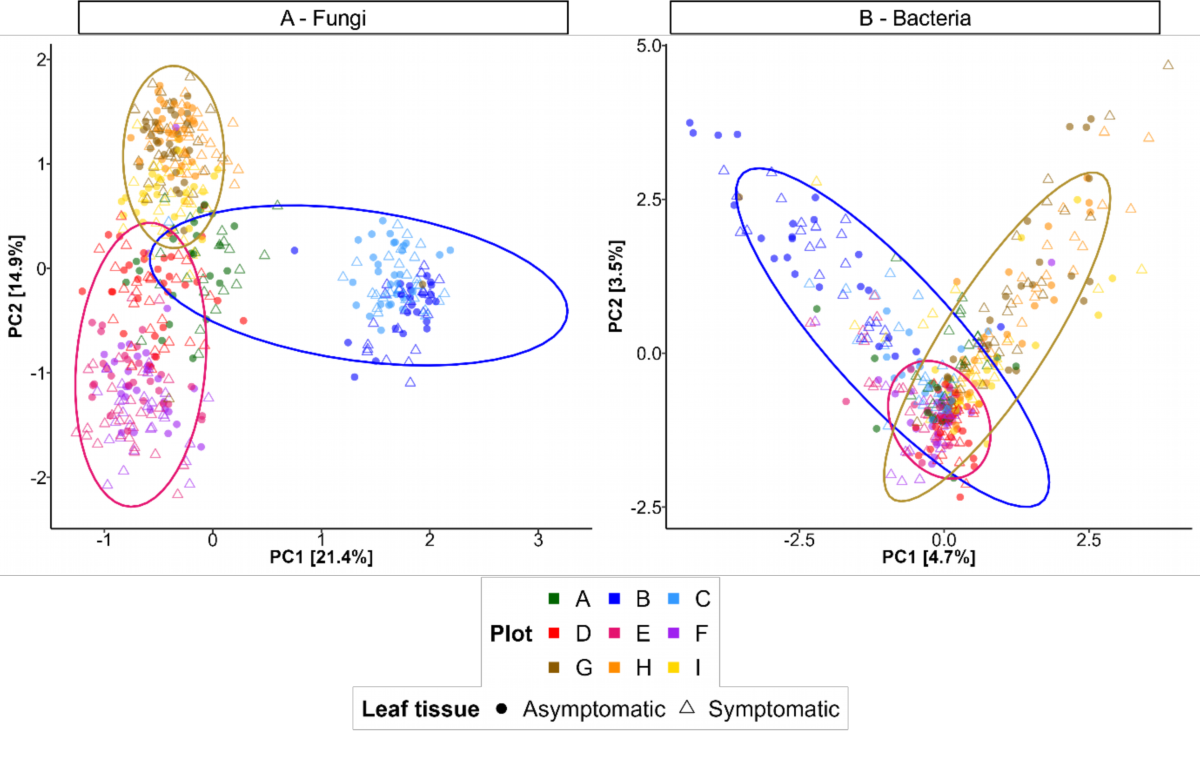


##### Figure S3 : Variation in microbial community composition across vineyard plots.

Compositional dissimilarities between (A) fungal and (B) bacterial communities of grapevine leaves, represented by a Principal Component Analysis (PCA). Samples collected from the same vineyard plot are represented with the same color. Samples belonging to the same region are enclosed within an ellipse (Occitanie in blue, Aquitaine in pink and Champagne in yellow, as in Fig. 1). Symbols indicate leaf tissue status: circles for asymptomatic leaves and triangles for downy mildew-symptomatic ones.


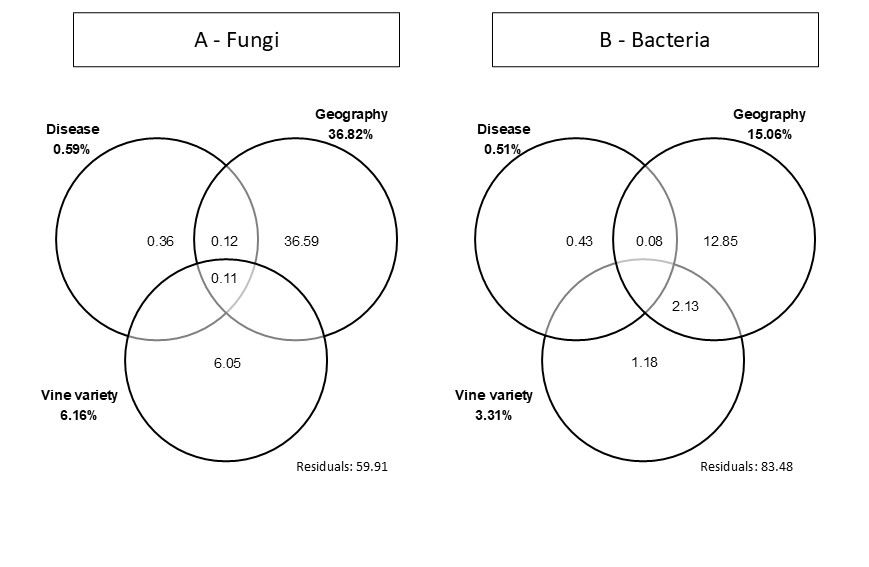


##### Figure S4 : Factors driving variation in microbial community composition according to variance partitioning.

The figure shows the factors influencing the composition of the (A) fungal and (B) bacterial communities. The environmental variables were grouped into three categories (Disease, Geography and Vine variety) to perform variance partitioning. The Venn diagrams show the percentage of variance explained by each group of variables, either individually or in combination with other groups.


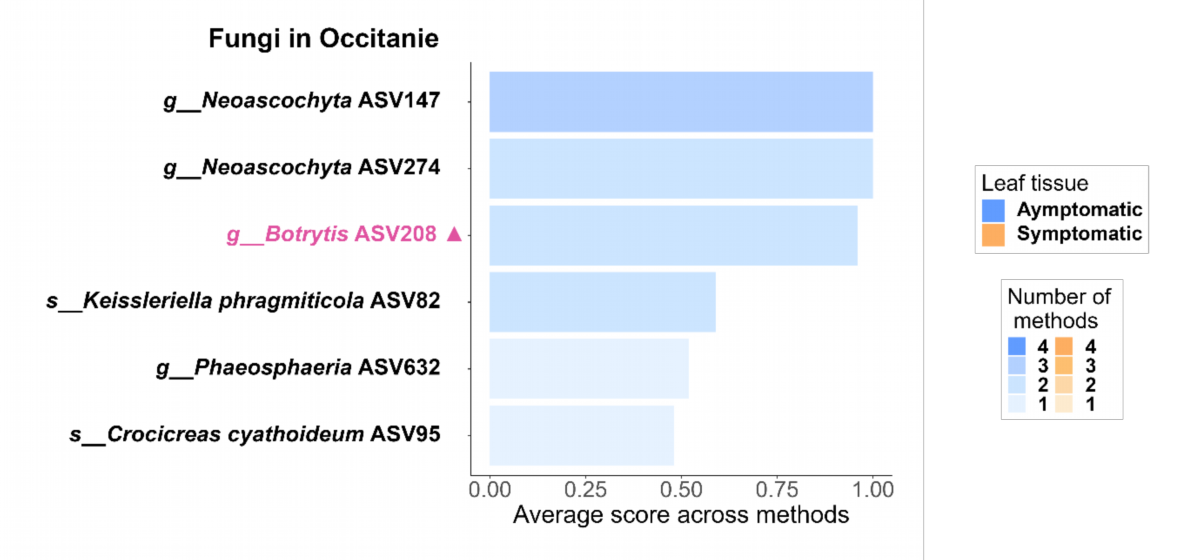


##### Figure S5 : Fungal ASVs in the Occitanie region that vary in abundance between asymptomatic and downy mildew-symptomatic grapevine leaf tissue.

For each condition (asymptomatic *vs.* symptomatic), we represented the ASVs that were significantly more abundant in that condition according to at least one method of differential abundance analysis (DAA). The four methods used to identify these ASVs are ANCOM-BC2 (Lin & Peddada, 2024a), MaAslin2 (Mallick *et al.*, 2021), LinDA (Zhou *et al.*, 2022), and ZicoSeq (Yang & Chen, 2022a). All analyses were conducted using only the data collected in the Occitanie region. Shades of blue and orange indicate the number of DAA methods that identified the ASV as differentially abundant (ranging from 1 to 4). ASVs belonging to basidiomycete yeasts are highlighted in green and marked with a dot while those known as foliar pathogens of grapevine are highlighted in pink and marked with a triangle. ASVs that do not meet either of these criteria are displayed in black without any specific symbol. ASVs that are also significant in the TITAN analysis (Baker *et al.*, 2023) are shown in bold. The y-axis provides information on the lowest taxonomic level at which each ASV was identified, including its prefix, assignment, and number of ASVs. The prefix abbreviations are *p* for phylum, *c* for class, *o* for order, *f* for family, *g* for genus, and *s* for species.


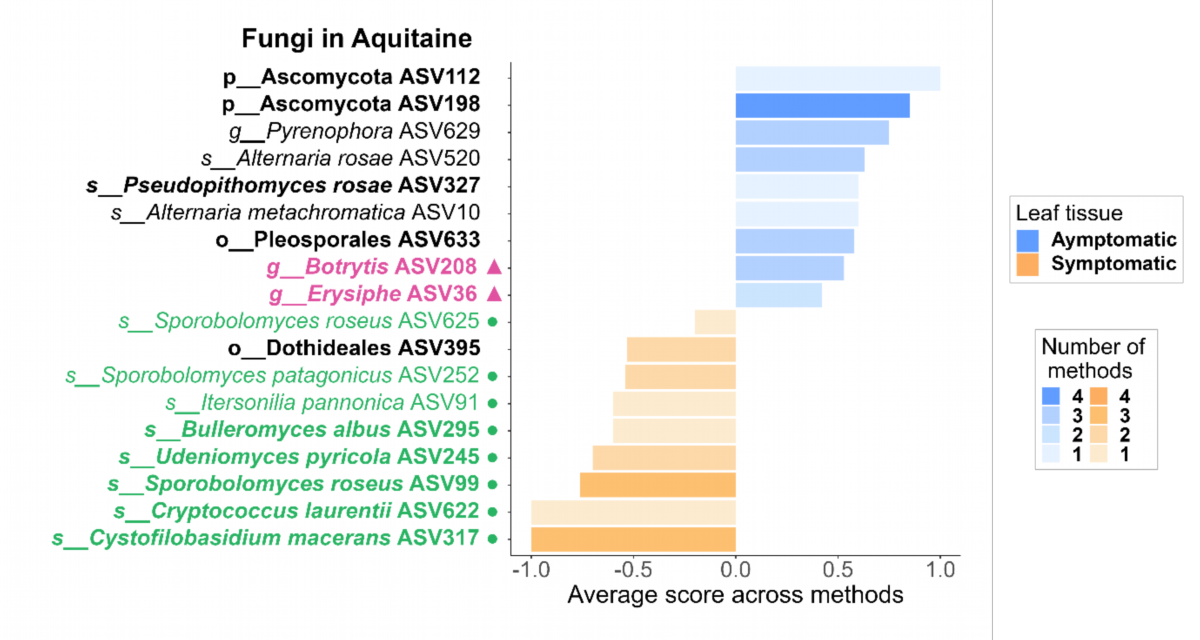


##### Figure S6 : Fungal ASVs in the Aquitaine region that vary in abundance between asymptomatic and downy mildew-symptomatic grapevine leaf tissue.

For each condition (asymptomatic *vs.* symptomatic), we represented the ASVs that were significantly more abundant in that condition according to at least one method of differential abundance analysis (DAA). The four methods used to identify these ASVs are ANCOM-BC2 (Lin & Peddada, 2024a), MaAslin2 (Mallick *et al.*, 2021), LinDA (Zhou *et al.*, 2022), and ZicoSeq (Yang & Chen, 2022a). All analyses were conducted using only the data collected in the Aquitaine region. Shades of blue and orange indicate the number of DAA methods that identified the ASV as differentially abundant (ranging from 1 to 4). ASVs belonging to basidiomycete yeasts are highlighted in green and marked with a dot while those known as foliar pathogens of grapevine are highlighted in pink and marked with a triangle. ASVs that do not meet either of these criteria are displayed in black without any specific symbol. ASVs that are also significant in the TITAN analysis (Baker *et al.*, 2023) are shown in bold. The y-axis provides information on the lowest taxonomic level at which each ASV was identified, including its prefix, assignment, and number of ASVs. The prefix abbreviations are *p* for phylum, *c* for class, *o* for order, *f* for family, *g* for genus, and *s* for species.


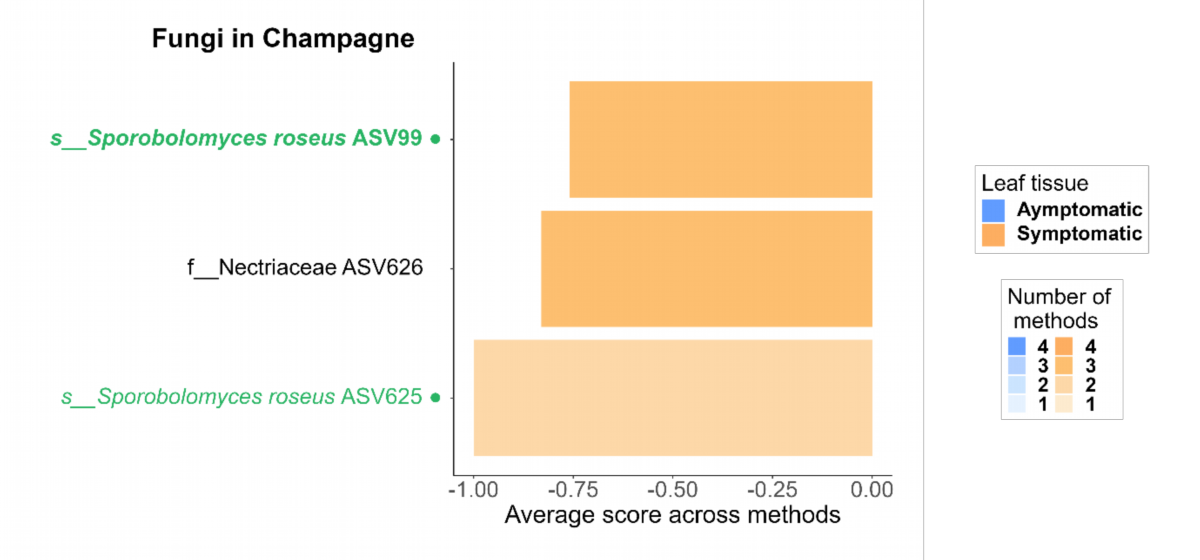


##### Figure S7 : Fungal ASVs in the Champagne region that vary in abundance between asymptomatic and downy mildew-symptomatic grapevine leaf tissue.

For each condition (asymptomatic *vs.* symptomatic), we represented the ASVs that were significantly more abundant in that condition according to at least one method of differential abundance analysis (DAA). The four methods used to identify these ASVs are ANCOM-BC2 (Lin & Peddada, 2024a), MaAslin2 (Mallick *et al.*, 2021), LinDA (Zhou *et al.*, 2022), and ZicoSeq (Yang & Chen, 2022a). All analyses were conducted using only the data collected in the Champagne region. Shades of blue and orange indicate the number of DAA methods that identified the ASV as differentially abundant (ranging from 1 to 4). ASVs belonging to basidiomycete yeasts are highlighted in green and marked with a dot while those known as foliar pathogens of grapevine are highlighted in pink and marked with a triangle. ASVs that do not meet either of these criteria are displayed in black without any specific symbol. ASVs that are also significant in the TITAN analysis (Baker *et al.*, 2023) are shown in bold. The y-axis provides information on the lowest taxonomic level at which each ASV was identified, including its prefix, assignment, and number of ASVs. The prefix abbreviations are *p* for phylum, *c* for class, *o* for order, *f* for family, *g* for genus, and *s* for species.


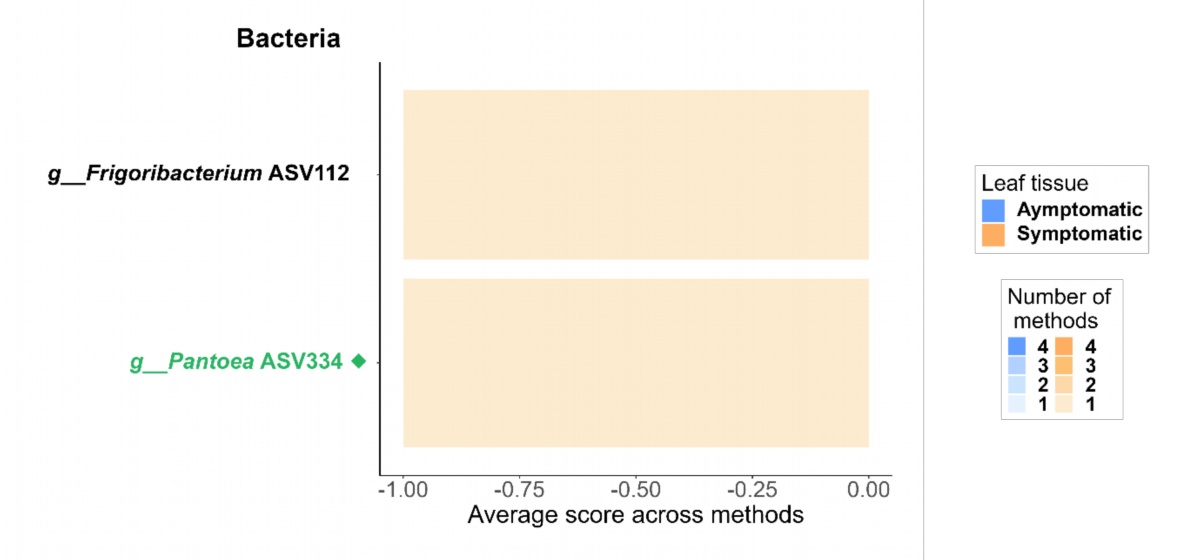


##### Figure S8 : Bacterial ASVs that vary in abundance between asymptomatic and downy mildew-symptomatic grapevine leaf tissue.

For each condition (asymptomatic *vs.* symptomatic), we represented the ASVs that were significantly more abundant in that condition according to at least one method of differential abundance analysis (DAA). The four methods used to identify these ASVs are ANCOM-BC2 (Lin & Peddada, 2024a), MaAslin2 (Mallick *et al.*, 2021), LinDA (Zhou *et al.*, 2022), and ZicoSeq (Yang & Chen, 2022a). All analyses were conducted at the national level (i.e., using the full dataset combining all three regions). Shades of blue and orange indicate the number of DAA methods that identified the ASV as differentially abundant (ranging from 1 to 4). Members of *Bacillus*, *Pantoea*, *Pseudomonas*, *Sphingomonas* and *Streptomyces* are shown in green followed by a diamond. The other ASVs are displayed in black without any specific symbol. ASVs that are also significant in the TITAN analysis (Baker *et al.*, 2023) are shown in bold. The y-axis provides information on the lowest taxonomic level at which each ASV was identified, including its prefix, assignment, and number of ASVs. The prefix abbreviations are *p* for phylum, *c* for class, *o* for order, *f* for family, *g* for genus, and *s* for species.


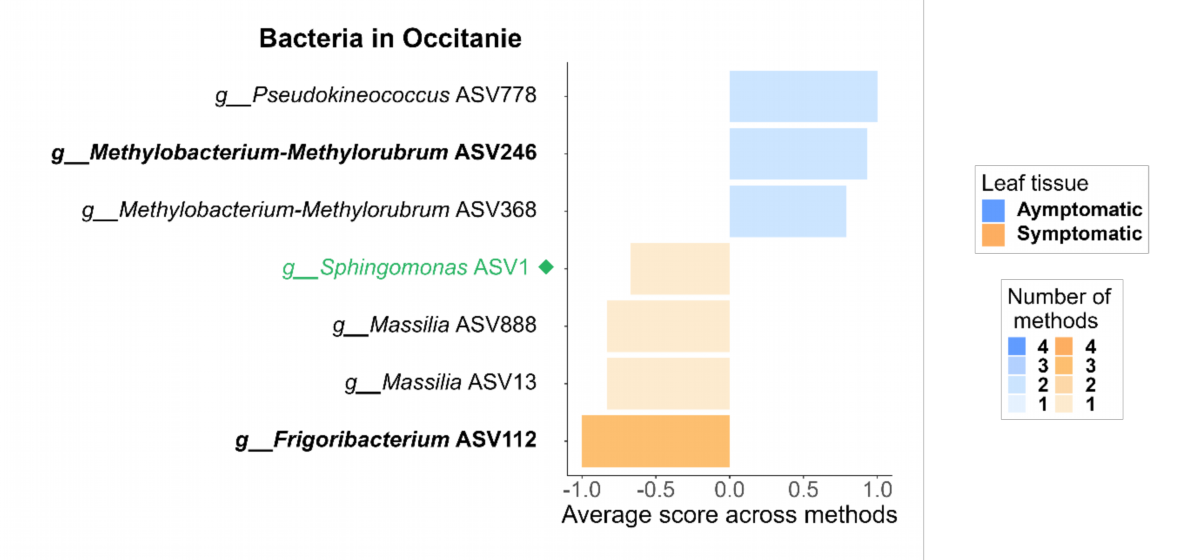


##### Figure S9 : Bacterial ASVs in the Occitanie region that vary in abundance between asymptomatic and downy mildew-symptomatic grapevine leaf tissue.

For each condition (asymptomatic *vs.* symptomatic), we represented the ASVs that were significantly more abundant in that condition according to at least one method of differential abundance analysis (DAA). The four methods used to identify these ASVs are ANCOM-BC2 (Lin & Peddada, 2024a), MaAslin2 (Mallick *et al.*, 2021), LinDA (Zhou *et al.*, 2022), and ZicoSeq (Yang & Chen, 2022a). All analyses were conducted using only the data collected in the Occitanie region. Shades of blue and orange indicate the number of DAA methods that identified the ASV as differentially abundant (ranging from 1 to 4). Members of *Bacillus*, *Pantoea*, *Pseudomonas*, *Sphingomonas* and *Streptomyces* are shown in green followed by a diamond. The other ASVs are displayed in black without any specific symbol. ASVs that are also significant in the TITAN analysis (Baker *et al.*, 2023) are shown in bold. The y-axis provides information on the lowest taxonomic level at which each ASV was identified, including its prefix, assignment, and number of ASVs. The prefix abbreviations are *p* for phylum, *c* for class, *o* for order, *f* for family, *g* for genus, and *s* for species.


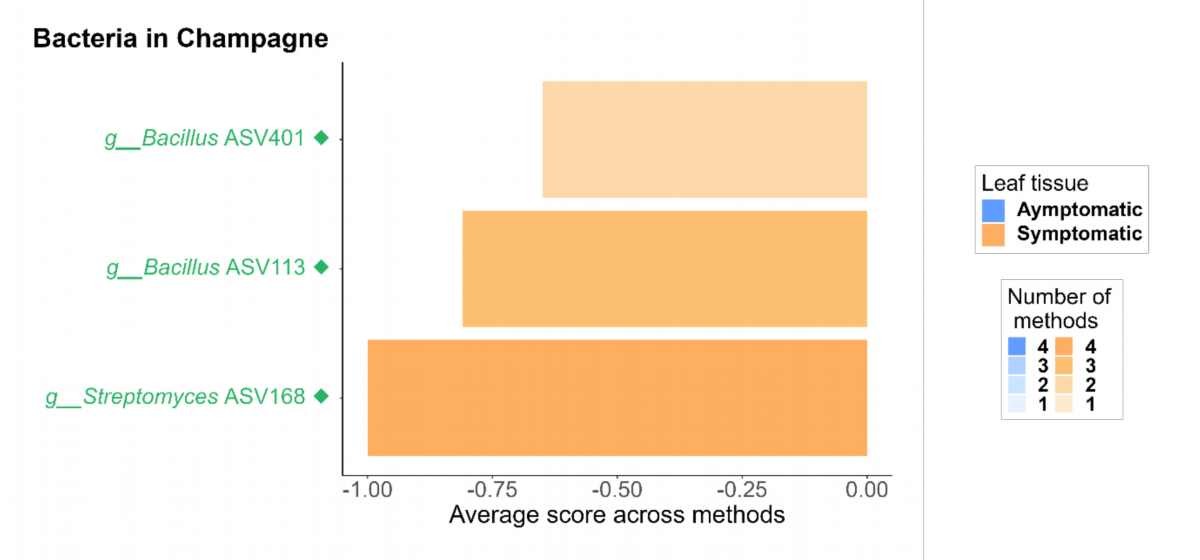


##### Figure S10 : Bacterial ASVs in the Champagne region that vary in abundance between asymptomatic and downy mildew-symptomatic grapevine leaf tissue.

For each condition (asymptomatic *vs.* symptomatic), we represented the ASVs that were significantly more abundant in that condition according to at least one method of differential abundance analysis (DAA). The four methods used to identify these ASVs are ANCOM-BC2 (Lin & Peddada, 2024a), MaAslin2 (Mallick *et al.*, 2021), LinDA (Zhou *et al.*, 2022), and ZicoSeq (Yang & Chen, 2022a). All analyses were conducted using only the data collected in the Champagne region. Shades of blue and orange indicate the number of DAA methods that identified the ASV as differentially abundant (ranging from 1 to 4). Members of *Bacillus*, *Pantoea*, *Pseudomonas*, *Sphingomonas* and *Streptomyces* are shown in green followed by a diamond. The other ASVs are displayed in black without any specific symbol. ASVs that are also significant in the TITAN analysis (Baker *et al.*, 2023) are shown in bold. The y-axis provides information on the lowest taxonomic level at which each ASV was identified, including its prefix, assignment, and number of ASVs. The prefix abbreviations are *p* for phylum, *c* for class, *o* for order, *f* for family, *g* for genus, and *s* for species.

## Supplementary tables

##### Table S1 : Factors driving variation in microbiota composition according to the redundancy analysis.

The table shows the variables kept by stepwise selection. The box is gray if the variable was not selected. The marginal effects of the variables and the p value were obtained using the ANOVA permutation test for redundancy analysis (RDA). The significance of the variables is as follows: ns (not significant), * (p<0.05), ** (p<0.01), *** (p<0.001).

|  |  | **Fungi** | | **Bacteria** | |
| --- | --- | --- | --- | --- | --- |
| **Group of variables** | **Selected variable** | **Marginal effect** | **p value** | **Marginal effect** | **p value** |
| **Disease** | **DNA concentration of *Plasmopara viticola* (qPCR data)** |  |  |  |  |
|  | **Leaf tissue (asymptomatic *vs.* downy mildew-symptomatic)** | 2.093 | 0.001 *** |  |  |
| **Geography** | **PCNM1** | 14.274 | 0.001 *** | 3.815 | 0.001 *** |
|  | **PCNM2** | 18.282 | 0.001 *** |  |  |
|  | **PCNM3** | 15.884 | 0.001 *** | 2.079 | 0.001 ** |
|  | **PCNM4** | 16.710 | 0.001 *** | 1.270 | 0.001 * |
|  | **PCNM5** | 6.315 | 0.001 *** | 2.271 | 0.001 *** |
|  | **Leaf** | 9.214 | 0.001 *** | 16.188 | 0.001 *** |
| **Variety** | **Grapevine variety** | 23.399 | 0.001 *** |  |  |

##### Table S2 : Factors driving variation in microbial α-diversity and load.

We analyzed the effects of leaf tissue (asymptomatic *vs.* downy mildew-symptomatic) and vineyard plots on microbial α-diversity and load. R²m and R²c represent the marginal and conditional determination coefficients of the model, respectively. N indicates the number of samples included. The significance of the variables is indicated as follows: ns (not significant), nonsignificant trend (0.1>p>0.05), * (p<0.05), ** (p<0.01), *** (p<0.001). To highlight which plots differed in microbial α-diversity and load, multiple comparisons were conducted and adjusted using the Bonferroni method. Plots (named A to I; Fig. 1) with the same superscript harbor microbiotas with similar diversity or load.

| **Model** | | | | **Tissue health condition**  **(Symptomatic *vs.* Asymptomatic)** | | **Geography**  **(Vineyard plot)** | |
| --- | --- | --- | --- | --- | --- | --- | --- |
| **Kingdom** | **N** | **R²c** | **R²m** | **p** | **Variation direction** | **p** | **Variation direction** |
| **Diversity index: Richness (Hill number, q = 0)** | | | | | | | |
| Fungi | 502 | 0.537 | 0.389 | ** | Asymptomatic > Symptomatic | *** | G^1^, I^1^, H^1^, B^1^ <  E^2^ < F^23^, C^23^, D^23^ < A^3^ |
| Bacteria | 390 | 0.293 | 0.170 | * | Asymptomatic > Symptomatic | *** | C^1^, A^1^, B^1^ < F^12^,  E^12^ < I^123^ < D^23^, G^23^ < H^3^ |
| **Diversity index: Shannon (Hill number, q = 1)** | | | | | | | |
| Fungi | 502 | 0.691 | 0.547 | ** | Asymptomatic > Symptomatic | *** | H^1^ < G^12^, I^12^ < B^2^ <  E^3^, F^3^ < A^34^, C^34^ < D^4^ |
| Bacteria | 390 | 0.406 | 0.259 | *** | Asymptomatic > Symptomatic | *** | B^1^ < A^12^, C^12^ < G^23^ <  E^234^, F^234^ < I^34^, H^34^ < D^4^ |
| **Diversity index: Simpson (Hill number, q = 2)** | | | | | | | |
| Fungi | 502 | 0.626 | 0.476 | ns | - | *** | H^1^ < G^12^, I^12^ < B^2^ <  E^3^, A^3^ < F^34^, C^34^ < D^4^ |
| Bacteria | 390 | 0.354 | 0.234 | *** | Asymptomatic > Symptomatic | *** | B^1^ < A^12^, C^12^ < G^123^ <  E^23^ < F^234^, I^234^ < H^34^ < D^4^ |
| **Microbial load** | | | | | | | |
| Fungi | 481 | 0.317 | 0.316 | *** | Symptomatic > Asymptomatic | *** | D^1^ < I^12^, G^12^ < F^123^ < C^23^,  E^23^ < H^3^ < A^4^, B^4^ |
| Bacteria | 369 | 0.412 | 0.398 | *** | Asymptomatic > Symptomatic | *** | G^1^, H^1^, B^1^, A^1^ <  I^12^ < C^123^ < E^234^ < D^34^ < F^4^ |

##### Table S3: List of all studies that investigated the relationship between the grapevine bacterial microbiota and downy mildew.

The table presents all studies that compared the composition of the grapevine bacterial microbiota between two conditions reflecting downy mildew (DM) abundance, referred to as High-DM vs Low-DM conditions. The columns "Microbial genera associated with Low-DM condition" and "Microbial genera associated with High-DM condition" list the microorganisms found to be more abundant under each condition. Bacterial genera highlighted in bold are of particular interest in the present study: *Bacillus*, *Pantoea*, *Streptomyces*, *Sphingomonas*, and *Pseudomonas*. Data sources and selection criteria for bacterial genera : Perazzolli *et al.* (2014) : from the main text ; Wicaksono *et al.* (2023) : from Figure 3-C ; Duret *et al.* (2025) : from Figure 2-A and Supplementary Figures 3-B and 3-C ; Fournier *et al.* (2025) : from Supplementary File S3 and S4, we retained bacterial genera found significant by at least one of the six methods, calculated an average association score for each genus, and selected the 20 genera with the highest average scores ; Present study: from Supplementary File S2 and S3, we retained bacterial genera found significant by at least one of the five methods, calculated an average association score for each genus, and selected the 20 genera with the highest average scores.

| **Study** | **Conditions reflecting downy mildew abundance (High-DM *vs* Low-DM)** | **Microbiota compared between the two conditions** | **Statistical method** | **Season** | **Country** | **Microbial genera associated with Low-DM condition** | **Microbial genera associated with High-DM condition** |
| --- | --- | --- | --- | --- | --- | --- | --- |
| Perazzolli *et al.* (2014) | High-DM : Relatively high disease severity  Low-DM : Relatively low disease severity | Phyllosphere microbiota | Spearman's rank correlations (P < 0.01) | June 2012 | Northern Italy (Udine,San Michele all'Adige | *Haemophilus, Kineosporia, Paracoccus, Porphyromonas, Roseomonas,* and *Swaminathania* | not specified |
| Wicaksono *et al.* (2023)  Focus on Alphaproteobacteria | High-DM :  Susceptible cultivars  (*V. vinifera* L. subsp. *Sylvestris*, *V. vinifera* L. subsp. *vinifera* 'Blauer Wildbacher' and 'Müller Thurgau', and *Vitis* × *alexanderi* Prince ex Jacques 'Isabella' )  Low-DM : Highly resistant species (*V. amurensis*, *V. riparia*) | Phyllosphere microbiota (leave area of 20 × 18 cm) | A method of differential abundance analysis (edgeR (Robinson *et al.*, 2010); P_adjusted_ < 0.1) | October 2014, when the berries are ripe for harvesting | Austria (Graz) | *Altererythrobacter, Devosia, Falshirhodobacter, Mesorhizobium, Novosphingobium, Ochrobactrum, Paracoccus, Pararhizobium, Rhizobium* | ***Sphingomonas*** |
| Duret *et al.* (2025) | High-DM : Susceptible cultivar (untreated Chardonnay)  Low-DM : Resistant cultivar (Voltis) | Rhizosphere microbiota | A method of differential abundance analysis (LEfSe (Segata *et al.*, 2011); LDA score ≥ 2.0 cutoff) | Flowering | France (Champagne region) | *Actinocorallia, Actinophytocola, Actinoplanes, Agromyces, Ahniella, Altererythrobacter, Arenimonas, Bradyrhizobium, Cellulosimicrobium, Chthoniobacter, Fluviicola, Haliangium, Hirschia, Kineosporia, Luteolibacter, Nordella, Ohtaekwangia,* ***Pantoea****, Phenylobacterium, Piscinabacter, Polaromonas, Promicromonospora, Reyranella, Rhizobacter, Roseimicrobium,* ***Sphingomonas****, Steroidobacter,* ***Streptomyces****, Terrimonas* | *Achromobacter, Arthrobacter, Chryseobacterium, Dyadobacter, Enterobacter, Microbacterium, Olivibacter, Paenarthrobacter, Pedobacter, Phyllobacterium,* ***Pseudomonas****, Rhizobium, Serratia, Sphingobacterium, Sphingopyxis, Stenotrophomonas, Taibaiella* |
| Duret *et al.* (2025) | High-DM : Susceptible cultivar (untreated Chardonnay)  Low-DM : Resistant cultivar (Voltis) | Rhizosphere microbiota | A method of differential abundance analysis (LEfSe (Segata *et al.*, 2011); LDA score ≥ 2.0 cutoff) | Veraison | France (Champagne region) | *Achromobacter, Bosea, Caenimonas, Glycomyces, Kineosporia, Lacunisphaera, Polaromonas, Promicromonospora, Rhizocola* | *Luteolibacter, Microbacterium, Niabella, Rhodoferax, Verrucomicrobium* |
| Duret *et al.* (2025) | High-DM : Untreated susceptible cultivar (Chardonnay)  Low-DM : Treated susceptible cultivar (Chardonnay) | Rhizosphere microbiota | A method of differential abundance analysis (LEfSe (Segata *et al.*, 2011); LDA score ≥ 2.0 cutoff) | Veraison | France (Champagne region) | *Bosea, Conexibacter, Phyllobacterium, Rhizocola, Serratia* | *Microbacterium, Neochlamydia, Paenarthrobacter, Paenibacillus, Rhodoferax, Sporocytophaga* |
| Fournier *et al.* (2025) | High-DM : High downy mildew incidence and severity over several years  Low-DM : Low downy mildew incidence and severity over several years | Topsoil (upper 5 cm) microbiota | Four methods for analyzing differential abundance (ANCOM-BC2 (Lin & Peddada, 2024b), MaAslin2 (Mallick *et al.*, 2021), LinDA (Zhou *et al.*, 2022), and ZicoSeq (Yang & Chen, 2022b)), and one Random Forest classification method (Wright & Ziegler, 2017) | From April 18 to May 2, 2022, and from April 11 to April 26, 2023; at the phenological stage of 2 to 3 leaves unfolded | France (Nouvelle-Aquitaine region) | *Actinotalea, Angustibacter, Aureimonas, Azohydromonas,* ***Bacillus****, Gemmatimonas, Kouleothrix, Massilia, Microlunatus, Modestobacter, Naasia, Noviherbaspirillum, Paenibacillus, Pseudarthrobacter, Roseomonas, Segetibacter, Solirubrobacter,* ***Streptomyces*** | *Acidibacter, Agromyces,* ***Bacillus****, Cellulomonas, Gaiella, Gemmatimonas, Haliangium,* *Hassallia, Iamia, Labilithrix, Microbacterium, Microlunatus, Nitrospira, Nocardioides, Polaromonas, Pseudonocardia, Rhodococcus, Rubrobacter, Solirubrobacter, Williamsia* |
|  | High-DM :  Relatively high concentration of *P. viticola* DNA in the topsoil  Low-DM :  Relatively low concentration of *P. viticola* DNA in the topsoil |  | TITAN (Baker *et al.*, 2023) |  |  |  |  |
| Fournier *et al.* (2025) | High-DM : High downy mildew incidence and severity over several years  Low-DM : Low downy mildew incidence and severity over several years | Phyllosphere microbiota | Four methods for analyzing differential abundance (ANCOM-BC2 (Lin & Peddada, 2024b), MaAslin2 (Mallick *et al.*, 2021), LinDA (Zhou *et al.*, 2022), and ZicoSeq (Yang & Chen, 2022b)), and one Random Forest classification method (Wright & Ziegler, 2017) | From April 18 to May 2, 2022, and from April 11 to April 26, 2023; at the phenological stage of 2 to 3 leaves unfolded | France (Nouvelle-Aquitaine region) | *Acidiphilium, Anaeromyxobacter, Arenimonas, Arthrobacter,* ***Bacillus****, Candidatus_Koribacter, Candidatus_Solibacter, Clostridium,*  *Dyadobacter, Flavisolibacter, Gaiella, Microlunatus, Nitrospira, Ohtaekwangia, Oligoflexus, Paenibacillus, Segetibacter, Skermanella, Terribacillus,*  *Terrimonas* | ***Bacillus****, Candidatus_Alysiosphaera, Fictibacillus, Flavisolibacter, Gaiella, Gemmatimonas, Ilumatobacter, Micromonospora, Mycobacterium, Nocardioides, Novosphingobium, Paracoccus, Pseudonocardia, Psychroglaciecola, Rubellimicrobium, Rubrobacter, Solirubrobacter,* ***Sphingomonas*** |
|  | High-DM :  Relatively high concentration of *P. viticola* DNA in the topsoil  Low-DM :  Relatively low concentration of *P. viticola* DNA in the topsoil |  | TITAN (Baker *et al.*, 2023) |  |  |  |  |
| Fournier *et al.* (2025) | High-DM : High downy mildew incidence and severity over several years  Low-DM : Low downy mildew incidence and severity over several years | Leaf endosphere microbiota | Four methods for analyzing differential abundance (ANCOM-BC2 (Lin & Peddada, 2024b), MaAslin2 (Mallick *et al.*, 2021), LinDA (Zhou *et al.*, 2022), and ZicoSeq (Yang & Chen, 2022b)), and one Random Forest classification method (Wright & Ziegler, 2017) | From April 18 to May 2, 2022, and from April 11 to April 26, 2023; at the phenological stage of 2 to 3 leaves unfolded | France (Nouvelle-Aquitaine region) | no significant genus | no significant genus |
|  | High-DM :  Relatively high concentration of *P. viticola* DNA in the topsoil  Low-DM :  Relatively low concentration of *P. viticola* DNA in the topsoil |  | TITAN (Baker *et al.*, 2023) |  |  |  |  |
| Present study | High-DM  : Downy mildew-symptomatic tissue  Low-DM  : Asymptomatic tissue | Leaf disc microbiota | Four methods for analyzing differential abundance (ANCOM-BC2 (Lin & Peddada, 2024b), MaAslin2 (Mallick *et al.*, 2021), LinDA (Zhou *et al.*, 2022), and ZicoSeq (Yang & Chen, 2022b)) | Between June 13 and July 27, 2018; at the pic of downy mildew epidemic | France (Occitanie, Nouvelle-Aquitaine and Champagne regions) | *Acidovorax, Acinetobacter, Altererythrobacter, Brevibacterium, Corynebacterium, Enhydrobacter, Hymenobacter, Iamia Intrasporangium, Knoellia, Marmoricola, Massilia, Methylobacterium-Methylorubrum, Microbacterium,* ***Pseudomonas****, Roseomonas, Saccharopolyspora, Serinibacter,* ***Sphingomonas****, Thermoactinomyces* | *Acidiphilium, Arthrobacter,* ***Bacillus****, Craurococcus-Caldovatus, Fictibacillus, Frigoribacterium, Janibacter, Okibacterium, Paenarthrobacter, Paenibacillus, Paenisporosarcina,* ***Pantoea****, Patulibacter, Pseudarthrobacter,* ***Pseudomonas****, Rhodococcus,* ***Sphingomonas****, Sporosarcina,* ***Streptomyces****, Xanthomonas* |
|  | High-DM : Relatively high concentration of *P. viticola* DNA in leaf tissue  Low-DM  : Relatively low concentration of *P. viticola* DNA in leaf tissue |  | TITAN (Baker *et al.*, 2023) |  |  |  |  |

##### Table S4: List of all studies that investigated the relationship between the grapevine fungal microbiota and downy mildew.

The table presents all studies that compared the composition of the grapevine fungal microbiota between two conditions reflecting downy mildew (DM) abundance, referred to as High-DM vs Low-DM conditions. The columns "Microbial genera associated with Low-DM condition" and "Microbial genera associated with High-DM condition" list the microorganisms found to be more abundant under each condition. Basidiomycetous yeasts are highlighted in bold, as they are of particular interest in the present study. Data sources and selection criteria for fungal genera : Perazzolli *et al.* (2014): from the main text ; Fournier *et al.* (2025): from Supplementary File S3 and S4, we retained fungal genera found significant by at least one of the six methods, calculated an average association score for each genus, and selected the 20 genera with the highest average scores ; Present study: from Supplementary File S2 and S3, we retained fungal genera found significant by at least one of the five methods, calculated an average association score for each genus, and selected the 20 genera with the highest average scores.

| **Study** | **Conditions reflecting downy mildew abundance (High-DM *vs* Low-DM)** | **Microbiota compared between the two conditions** | **Statistical method** | **Season** | **Country** | **Microbial genera associated with Low-DM condition** | **Microbial genera associated with High-DM condition** |
| --- | --- | --- | --- | --- | --- | --- | --- |
| Perazzolli *et al.* (2014) | High-DM : Relatively high disease severity  Low-DM : Relatively low disease severity | Phyllosphere microbiota | Spearman's rank correlations (P < 0.01) | June 2012 | Northern Italy (Udine,San Michele all'Adige | *Chalastospora, Claviceps, Epicoccum, Exophiala, and Teratosphaeria* | not specified |
| Fournier *et al.* (2025) | High-DM : High downy mildew incidence and severity over several years  Low-DM : Low downy mildew incidence and severity over several years | Topsoil (upper 5 cm) microbiota | Four methods for analyzing differential abundance (ANCOM-BC2 (Lin & Peddada, 2024b), MaAslin2 (Mallick *et al.*, 2021), LinDA (Zhou *et al.*, 2022), and ZicoSeq (Yang & Chen, 2022b)), and one Random Forest classification method (Wright & Ziegler, 2017) | From April 18 to May 2, 2022, and from April 11 to April 26, 2023; at the phenological stage of 2 to 3 leaves unfolded | France (Nouvelle-Aquitaine region) | *Alternaria, Articulospora, Ascochyta, Aspergillus, Atrocalyx, Cladosporium, Coniochaeta, Didymella, Lectera, Mollisia, Mortierella,* ***Papiliotrema****, Paraphoma, Penicillium, Pseudeurotium, Spizellomyces, Stemphylium, Tetracladium, Trichoderma, Volutella* | *Albifimbria, Aureobasidium,* ***Bensingtonia****, Blumeria, Cadophora, Cladosporium,* ***Cystofilobasidium****, Emericellopsis, Fusarium, Fusicolla, Gliomastix, Leptospora, Lophiostoma, Lycoperdon, Mortierella, Neoerysiphe, Penicillium, Phaeotremella, Solicoccozyma, Tetracladium, Thelonectria,* ***Vishniacozyma****, Vuilleminia* |
|  | High-DM :  Relatively high concentration of *P. viticola* DNA in the topsoil  Low-DM :  Relatively low concentration of *P. viticola* DNA in the topsoil |  | TITAN (Baker *et al.*, 2023) |  |  |  |  |
| Fournier *et al.* (2025) | High-DM : High downy mildew incidence and severity over several years  Low-DM : Low downy mildew incidence and severity over several years | Phyllosphere microbiota | Four methods for analyzing differential abundance (ANCOM-BC2 (Lin & Peddada, 2024b), MaAslin2 (Mallick *et al.*, 2021), LinDA (Zhou *et al.*, 2022), and ZicoSeq (Yang & Chen, 2022b)), and one Random Forest classification method (Wright & Ziegler, 2017) | From April 18 to May 2, 2022, and from April 11 to April 26, 2023; at the phenological stage of 2 to 3 leaves unfolded | France (Nouvelle-Aquitaine region) | *Angustimassarina, Aspergillus, Atrocalyx,* ***Buckleyzyma****, Curvularia, Cytospora, Didymella,* ***Filobasidium****, Gibberella, Golovinomyces,* ***Itersonilia****, Jattaea,* ***Leucosporidium****, Peniophora, Plagiostoma, Plectosphaerella, Pseudopeziza,* ***Rhodotorula****, Stereum,* ***Vishniacozy*ma** | *Articulospora, Aspergillus, Chloridium, Dactylonectria, Exophiala, Fomes, Fusarium, Knufia, Leptospora, Metacordyceps, Monocillium, Mortierella, Penicillium, Peniophora, Solicoccozyma, Thelonectria, Trichoderma,* ***Udeniomyce****s,* ***Vishniacozyma****, Volucrispora* |
|  | High-DM :  Relatively high concentration of *P. viticola* DNA in the topsoil  Low-DM :  Relatively low concentration of *P. viticola* DNA in the topsoil |  | TITAN (Baker *et al.*, 2023) |  |  |  |  |
| Fournier *et al.* (2025) | High-DM : High downy mildew incidence and severity over several years  Low-DM : Low downy mildew incidence and severity over several years | Leaf endosphere microbiota | Four methods for analyzing differential abundance (ANCOM-BC2 (Lin & Peddada, 2024b), MaAslin2 (Mallick *et al.*, 2021), LinDA (Zhou *et al.*, 2022), and ZicoSeq (Yang & Chen, 2022b)), and one Random Forest classification method (Wright & Ziegler, 2017) | From April 18 to May 2, 2022, and from April 11 to April 26, 2023; at the phenological stage of 2 to 3 leaves unfolded | France (Nouvelle-Aquitaine region) | *Aspergillus, Cercophora,* ***Cystofilobasidium****, Fusarium, Fusicolla, Gnomonia, Humicola, Malassezia, Mrakiella, Stephanonectria, Tetracladium,* ***Vishniacozyma*** | *Blumeria, Botrytis, Clonostachys, Exophiala, Hypomyces,* ***Itersonilia****, Mortierella, Operculomyces, Podosphaera, Pyrenochaetopsis, Schizothecium, Tomentella, Trichoderma,* ***Udeniomyces*** |
|  | High-DM :  Relatively high concentration of *P. viticola* DNA in the topsoil  Low-DM :  Relatively low concentration of *P. viticola* DNA in the topsoil |  | TITAN (Baker *et al.*, 2023) |  |  |  |  |
| Present study | High-DM  : Downy mildew-symptomatic tissue  Low-DM  : Asymptomatic tissue | Leaf disc microbiota | Four methods for analyzing differential abundance (ANCOM-BC2 (Lin & Peddada, 2024b), MaAslin2 (Mallick *et al.*, 2021), LinDA (Zhou *et al.*, 2022), and ZicoSeq (Yang & Chen, 2022b)) | Between June 13 and July 27, 2018; at the pic of downy mildew epidemic | France (Occitanie, Nouvelle-Aquitaine and Champagne regions) | *Alternaria, Angustimassarina, Antrodia, Blumeria, Botrytis, Coprinopsis,* ***Dioszegia****,* ***Genolevuria****, Hyphodermella,* ***Itersonilia****, Neoascochyta, Peniophora, Pseudopithomyces, Pyrenophora, Sigarispora, Sporormiella, Stemphylium,* ***Symmetrospora****, Taphrina, Vuilleminia* | ***Bulleromyces****, Cladosporium,* ***Cryptococcus****,* ***Curvibasidium*** ***Cystofilobasidium, Filobasidium****,* ***Holtermanniella****, Mycosphaerella, Neoascochyta, Septoria,* ***Sporobolomyces****,* ***Udeniomyces****,* ***Vishniacozyma*** |
|  | High-DM : Relatively high concentration of *P. viticola* DNA in leaf tissue  Low-DM  : Relatively low concentration of *P. viticola* DNA in leaf tissue |  | TITAN (Baker *et al.*, 2023) |  |  |  |  |

**References**

Baker ME, King RS, Kahle [aut D, cph, cre, 2023. TITAN2: Threshold Indicator Taxa Analysis.

Duret M, Wallner A, Besaury L, Aziz A, 2025. Diversity and functional features of the root-associated bacteriome are dependent on grapevine susceptibility to Plasmopara viticola. *Environmental Microbiome* **20**, 30.

Fournier P, Pellan L, Jaswa A *et al.*, 2025. Revealing microbial consortia that interfere with grapevine downy mildew through microbiome epidemiology. *Environmental Microbiome* **20**, 37.

Lin H, Peddada SD, 2024a. Multigroup analysis of compositions of microbiomes with covariate adjustments and repeated measures. *Nature Methods* **21**, 83–91.

Lin H, Peddada SD, 2024b. Multigroup analysis of compositions of microbiomes with covariate adjustments and repeated measures. *Nature Methods* **21**, 83–91.

Mallick H, Rahnavard A, McIver LJ *et al.*, 2021. Multivariable association discovery in population-scale meta-omics studies. *PLOS Computational Biology* **17**, e1009442.

Perazzolli M, Antonielli L, Storari M *et al.*, 2014. Resilience of the Natural Phyllosphere Microbiota of the Grapevine to Chemical and Biological Pesticides (HL Drake, Ed,). *Applied and Environmental Microbiology* **80**, 3585–3596.

Robinson MD, McCarthy DJ, Smyth GK, 2010. edgeR: a Bioconductor package for differential expression analysis of digital gene expression data. *Bioinformatics* **26**, 139–140.

Segata N, Izard J, Waldron L *et al.*, 2011. Metagenomic biomarker discovery and explanation. *Genome Biology* **12**, R60.

Wicaksono WA, Morauf C, Müller H, Abdelfattah A, Donat C, Berg G, 2023. The mature phyllosphere microbiome of grapevine is associated with resistance against *Plasmopara viticola*. *Frontiers in Microbiology* **14**.

Wright MN, Ziegler A, 2017. ranger: A Fast Implementation of Random Forests for High Dimensional Data in C++ and R. *Journal of Statistical Software* **77**, 1–17.

Yang L, Chen J, 2022a. A comprehensive evaluation of microbial differential abundance analysis methods: current status and potential solutions. *Microbiome* **10**, 130.

Yang L, Chen J, 2022b. A comprehensive evaluation of microbial differential abundance analysis methods: current status and potential solutions. *Microbiome* **10**, 130.

Zhou H, He K, Chen J, Zhang X, 2022. LinDA: linear models for differential abundance analysis of microbiome compositional data. *Genome Biology* **23**, 95.
